# NSD2 Coordinates the Neurogenic‐to‐Gliogenic Transition via H3K36me2‐Dependent Activation of the EGFR‐ERK Pathway

**DOI:** 10.1002/advs.76695

**Published:** 2026-07-27

**Authors:** Hanxue Chen, Mengyuan Li, Lin Hou, Bin Yin, Boqin Qiang, Ran Gao, Pengcheng Shu, Xiaozhong Peng

**Affiliations:** ^1^ State Key Laboratory of Common Mechanism Research for Major Diseases, Department of Biochemistry & Molecular Biology, Institute of Basic Medical Sciences & School of Basic Medicine Chinese Academy of Medical Sciences & Peking Union Medical College Beijing China; ^2^ National Human Diseases Animal Model Resource Center, Institute of Laboratory Animal Science Chinese Academy of Medical Sciences & Peking Union Medical College Beijing China; ^3^ State Key Laboratory of Respiratory Health and Multimorbidity, Institute of Laboratory Animal Science Chinese Academy of Medical Sciences & Peking Union Medical College Beijing China

**Keywords:** ERK, H3K36me2, intellectual disability, neocortex, NSD2

## Abstract

Haploinsufficiency of the histone methyltransferase NSD2 is a major cause of Wolf‐Hirschhorn syndrome (WHS) and the related Rauch‐Steindl syndrome (RAUST), both of which exhibit microcephaly and intellectual disability. However, the precise role of NSD2 in brain development remains unclear. Here, we identify NSD2 as a pivotal epigenetic regulator orchestrating the transition from neurogenesis to gliogenesis in the developing mouse neocortex. Conditional knockout of *Nsd2* severely impairs astrocyte production in late embryogenesis, while its overexpression promotes astrocytic fate. Integrated epigenomic and transcriptomic analyses reveal that NSD2 deposits the activating histone mark H3K36me2 directly at the *Egfr* promoter, sustaining EGFR expression and downstream ERK signaling—a pathway essential for gliogenesis. Pharmacological activation of ERK phosphorylation rescues the astrogliogenesis defects both in vitro and in vivo. Notably, *Nsd2*‐deficient mice exhibit significant deficits in learning and memory. Our findings define an NSD2‐H3K36me2‐EGFR‐ERK axis that drives cortical gliogenesis and provide mechanistic insights into the potential contribution of NSD2 deficiency to neurodevelopmental abnormalities.

## Graphical abstract

NSD2 coordinates the neurogenic‐to‐gliogenic transition in the developing neocortex through H3K36me2‐dependent activation of EGFR–ERK signaling. Loss of NSD2 disrupts astroglial and oligodendroglial development, whereas ERK activation rescues gliogenic defects in vitro and in vivo. These findings establish an NSD2–H3K36me2–EGFR–ERK axis linking epigenetic regulation of gliogenesis to neurodevelopmental dysfunction.

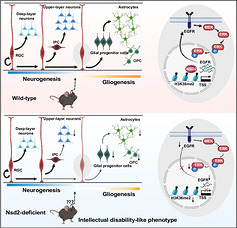

## 1 Introduction

The mammalian cerebral cortex, a structure of remarkable cellular complexity, is assembled through precisely coordinated sequences of neurogenesis and gliogenesis. This process hinges on the dynamic interplay between extrinsic signaling cues and cell‐intrinsic epigenetic programs, which together specify the identities and connectivity of diverse neuronal and glial populations [1, 2, 3]. Radial glial cells (RGCs), which reside in the ventricular zone (VZ), serve as primary neural progenitors. They initially generate excitatory neurons before undergoing a developmental competence shift—the neurogenic‐to‐gliogenic switch—to produce astrocytes and oligodendrocytes [4, 5]. This switch is confined to a narrow temporal window, peaking around embryonic day 16 (E16) in mice, with approximately 15%–20% of RGCs committing to the glial lineage [6, 7]. It is governed by conserved signaling pathways such as JAK‐STAT, Bone Morphogenetic Protein (BMP), NOTCH, and Fibroblast Growth Factor (FGF) [8, 9, 10].

Epigenetic regulation, particularly histone methylation, plays an equally critical role in fate determination [11, 12, 13]. Methylation of histone H3 lysine 36 (H3K36) is intricately linked to transcriptional regulation and is indispensable for neural development [14, 15]. Distinct enzymatic machineries mediate these modifications: the Nuclear receptor‐binding SET domain (NSD) family (NSD1, NSD2, and NSD3), ASH1L, SETMAR, and SMYD2 primarily catalyze H3K36me1/2, whereas H3K36me3 is predominantly established by SETD2 and SETD5 [15]. While several H3K36 methyltransferases, including NSD1, ASH1L, SETD5, and SETD2, have established roles in neuronal development and are implicated in neurodevelopmental disorders [6, 16, 17, 18], the function of NSD2 (also known as MMSET/WHSC1) in cortical development remains largely unexplored. This knowledge gap is striking given that haploinsufficiency of NSD2 is considered the primary genetic cause of Wolf‐Hirschhorn Syndrome (WHS), which is characterized by severe growth retardation, seizures, intellectual disability, and distinctive craniofacial abnormalities [19, 20]. Recently, loss‐of‐function variants in *NSD2* have also been linked to Rauch–Steindl syndrome (RAUST), which presents distinct facial and clinical features but similarly involves growth retardation, microcephaly, and intellectual disability (often mild) [21, 22]. The pronounced cognitive deficits in both WHS and RAUST patients strongly suggest a crucial, yet undefined, role for NSD2 in brain development.

In this study, we employed a combinatorial approach using *Emx1‐*Cre and *hGFAP‐*Cre mediated conditional knockout mice, complemented by in utero electroporation‐based gain‐of‐function assays, to systematically dissect the role of NSD2 in neocortical development. We demonstrate that NSD2 promotes the neurogenesis‐to‐gliogenesis transition in radial glial cells and facilitates the efficient generation of astroglial and oligodendroglial lineage cells during late cortical development. Mechanistically, we delineate a regulatory pathway whereby NSD2 catalyzes H3K36me2 deposition at promoters of key MAPK‐ERK signaling components, including *Egfr* and *Fgfr2*, to sustain pathway output. Furthermore, *Nsd2*‐deficient mice exhibited impaired learning and memory behaviors. Our study thus uncovers a precise epigenetic mechanism controlling cortical gliogenesis and provides a novel cellular framework for understanding NSD2‐related abnormal neurodevelopment.

## 2 Results

### 2.1 NSD2 is Broadly Expressed With a Late Embryonic Peak Coinciding With Gliogenesis

To elucidate the physiological function of NSD2 in cortical development, we first analyzed the expression landscape of the NSD family (*Nsd1, Nsd2, and Nsd3*) in the developing mouse cortex using BGI STOmicsDB and published single‐cell transcriptomic datasets [23]. While all three members were detected, *Nsd2* exhibited the highest overall expression level (Figure S1A). Temporal trajectory analysis revealed a dynamic shift: *Nsd1* was abundant during early‐to‐mid neurogenesis (E14.5–E16.5), whereas *Nsd2* expression increased markedly and became predominant after E17.5 (Figure S1B), coinciding with the onset of gliogenesis. Cell‐type‐specific analysis confirmed robust *Nsd2* expression across diverse lineages, including apical progenitors (APs), intermediate progenitors (IPs), deep‐ and upper‐layer cortical projection neurons (PNs), astrocytes, and oligodendrocytes (Figure S1C–E). Western blot and immunofluorescence validated widespread NSD2 protein expression throughout the developing neocortex (Figure S1F,G). Collectively, these data position NSD2 as a widely expressed methyltransferase whose upregulation temporally aligns with the gliogenic phase of corticogenesis.

### 2.2 Loss of NSD2 Impairs Cortical Growth and Astrogenesis

To define the function of NSD2 in neocortical development, we generated *Nsd2* conditional knockout (cKO) models by crossing *Nsd2* ^fl/fl^ mice with *Emx1‐*Cre and *hGFAP‐*Cre driver lines (Figure S2A). Specifically, *Emx1‐*Cre activity commences at embryonic day 9.5 (E9.5), targeting dorsal RGCs and their broad progeny, including cells within the hippocampus [24, 25]. In contrast, *hGFAP‐*Cre activity is initiated later, at approximately E12.5, predominantly targeting the dorsal neocortex and hippocampus, and persists in astrocytes into adulthood [25, 26] (Figure S2B). Given its earlier onset and broader lineage coverage, we therefore primarily focused our analyses on *Nsd2* ^fl/fl^; *Emx1‐*Cre mice (hereafter referred to as *Nsd2* ^Emx1−cKO^).

**FIGURE 1:**
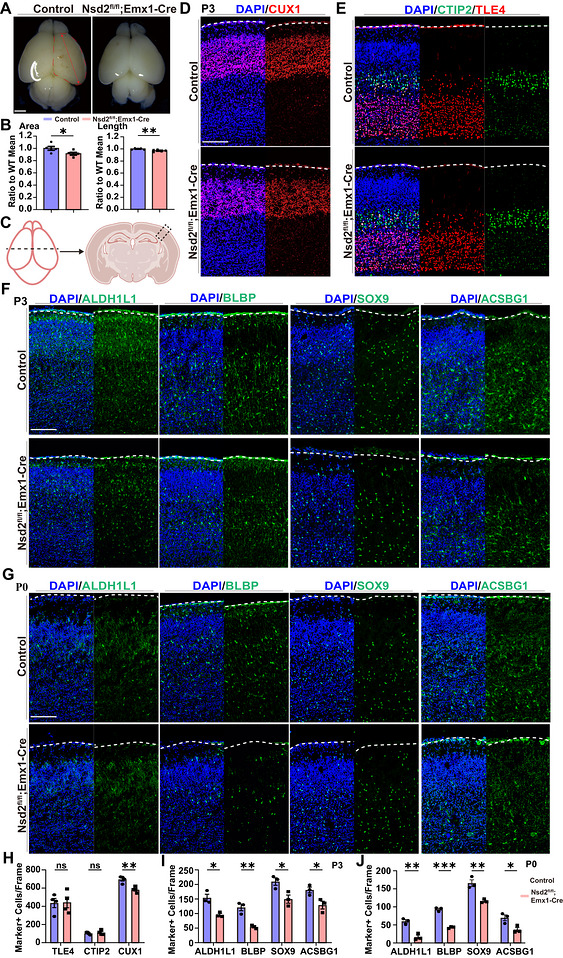
Knockout of *Nsd2* reduces the number of upper‐layer neurons and astrocytes. (A) Stereomicroscopic images of P3 brains following PFA perfusion. Red dashed lines and arrows indicate the measured area and length of the telencephalon, respectively. Scale bar, 2 mm. (B) Quantitative analysis of the cortical area and length shown in (A) (*n* = 5). (C) Schematic diagram illustrating the anatomical region of P3 coronal sections used for all analyses in this study. (D and E) Immunostaining for the cortical projection neuron markers CUX1, CTIP2, and TLE4 in the neocortex at P3. Scale bar, 200 µm. (F and G) Immunostaining for the astrocyte markers ALDH1L1, BLBP, SOX9, and ACSBG1 in the neocortex at P3 (F) and P0 (G). Scale bar, 200 µm. (H) Quantitative analysis of TLE4^+^, CTIP2^+^, and CUX1^+^ neurons in the neocortex at P3. (I and J) Quantitative analysis of ALDH1L1^+^, BLBP^+^, SOX9^+^, and ACSBG1^+^ astrocytes in the neocortex at P3 (I) and P0 (J). For all quantitative analyses of P3 and P0 sections, positive cells were counted within a rectangular region of interest (ROI) of 500 µm (width) × 1000 µm (height) (*n* = 3 or 4 biological replicates). Data are presented as mean ± SEM. Unpaired Student's *t*‐test or Welch's *t*‐test was used as appropriate. Detailed statistical information is provided in Table S2. *p < 0.05, **p < 0.01, ***p < 0.001, ****p < 0.0001.

Following genotypic confirmation, knockout efficiency in the dorsal forebrain was confirmed by Western blot and immunofluorescence, demonstrating efficient depletion of NSD2 protein in *Nsd2* ^Emx1−cKO^ mice (Figure S2C–E). Although born at expected Mendelian ratios, mutant mice exhibited significant postnatal growth retardation from week 1 to 12, as evidenced by reduced body weight and length compared to littermate controls (Figure S2F–H). At postnatal day 3 (P3), *Nsd2* ^Emx1−cKO^ mutant brains showed reduced telencephalic surface area and cortical length (Figure 1A,B), indicating impaired cortical development.

Immunofluorescence analysis of P3 cortices revealed largely preserved laminar organization. However, we observed a mild but significant reduction in upper‐layer CUX1^+^ neurons, while deep‐layer (CTIP2^+^, TLE4^+^) neurons were unaffected (Figure 1C–E,H). Given the temporal surge of NSD2 expression during the peak of gliogenesis, we next examined the astrocytic lineage (Figure 1F). Strikingly, the density of astrocytes, marked by ALDH1L1, BLBP, SOX9, and ACSBG1 [27], was severely reduced in P3 *Nsd2* ^Emx1−cKO^ cortices (Figure 1F,I). This astrocyte deficit was even more pronounced at P0 (Figure 1G,J). Similar cortical growth defects, as well as neuronal and astrocytic phenotypes, were recapitulated in *Nsd2* ^hGFAP−cKO^ mice (Figure S3), confirming the robustness of the findings. Collectively, these results demonstrate that NSD2 regulates cortical development, with a predominant effect on the generation and development of astrocytes during late embryogenesis.

### 2.3 *Nsd2* Deficiency Impairs Gliogenic Progression of Cortical RGCs

We next examined whether the astrocyte depletion resulted from aberrant apoptosis. TUNEL staining showed an increase in TUNEL^+^ cells in the dorsal cortex of *Nsd2* ^Emx1−cKO^ mice during late embryogenesis (Figure S4A,B). To determine whether astrocyte‐lineage cells were affected, we performed co‐immunostaining for activated Caspase‐3 and BLBP at P3. Compared with controls, *Nsd2* ^Emx1−cKO^ mice showed significant increases in both total Caspase‐3^+^ cells and Caspase‐3^+^BLBP^+^ cells (Figure S4C–E). Notably, apoptotic cells were mainly enriched in the white matter, whereas few were detected in the cortical plate despite the marked astrocyte reduction in this region (Figure S4C). These findings suggest that increased apoptosis contributes to astrocyte loss in specific regions but is insufficient to fully explain the cortical phenotype, which more likely results from impaired fate transition and/or altered progenitor proliferation.

We therefore focused on the critical gliogenic window (E16‐E17), as lineage‐tracing studies using Mosaic Analysis with Double Markers (MADM) indicate that a subpopulation of RGCs switches from neurogenic to gliogenic fate within a narrow temporal window, peaking at E16 [6, 7]. To determine whether the astrocyte deficit arises from an aberrant fate transition, we characterized RGC and intermediate progenitor (IP) populations at E12.5, E14.5, and E16.5 by immunofluorescence staining for PAX6 and TBR2, respectively (Figure S5). At E12.5 and E14.5, the numbers of PAX6^+^ RGCs and TBR2^+^ IPs were comparable between control and *Nsd2* ^Emx1−cKO^ cortices (Figure S5A–E). These stages overlap with deep‐layer CTIP2^+^ neuron production, consistent with the unchanged CTIP2^+^ neuron abundance in mutant cortices (Figure 1H). In contrast, at E16.5, although PAX6^+^ RGC density remained unchanged, TBR2^+^ IPs and PAX6^+^TBR2^+^ transitional progenitors were significantly reduced in *Nsd2* ^Emx1−cKO^ cortices (Figure S5F–I). Comparable findings were obtained in *Nsd2* ^hGFAP−cKO^ mice (Figure S5J–L), indicating a defective RGC‐to‐IP transition that likely underlies the subsequent reduction in upper‐layer neurons.

**FIGURE 2:**
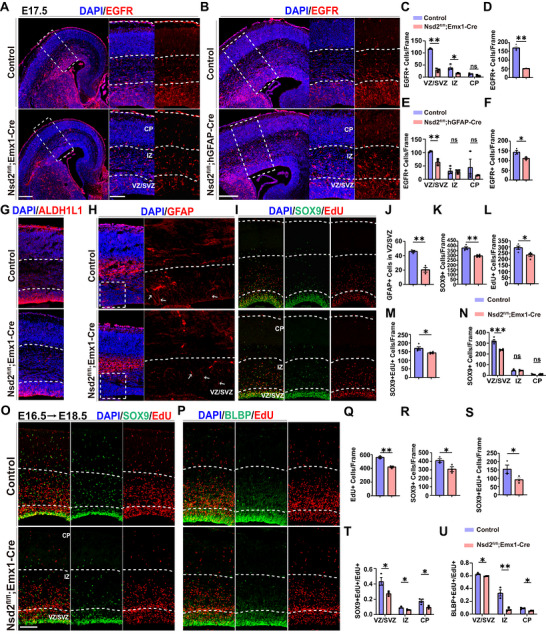
NSD2 Deletion Impairs the Transition of Radial Glial Cells to Gliogenic Progenitors. (A) Immunofluorescence analysis of EGFR^+^ gliogenic progenitors at E17.5 in *Nsd2* ^Emx1−cKO^ mice. (B) Immunofluorescence analysis of EGFR^+^ gliogenic progenitors at E17.5 in *Nsd2* ^hGFAP−cKO^ mice. (C, D) Distribution of EGFR^+^ cells across cortical zones (VZ/SVZ, IZ, and CP) (C) and quantification of the total EGFR^+^ cell number (D) in Figure 2A. (E, F) Distribution of EGFR^+^ cells across cortical zones (VZ/SVZ, IZ, and CP) (E) and quantification of the total EGFR^+^ cell number (F) in Figure 2B. (G) Immunofluorescence analysis of ALDH1L1^+^ cells at E17.5 in *Nsd2* ^Emx1‐cKO^ mice. (H) Quantification of total GFAP^+^ cells in the E17.5 *Nsd2* ^Emx1−cKO^ neocortex; White arrows indicate representative GFAP^+^ cells that were quantified. (I) Representative immunofluorescence images showing colocalization of the glial lineage marker SOX9 and the proliferation marker EdU at E17.5 following a 2 h EdU pulse‐labeling experiment. (J) Quantification of GFAP^+^ cells specifically within the VZ/SVZ at E17.5. (K–N) Quantitative analysis of total SOX9^+^ cells (K), total EdU^+^ cells (L), proliferating SOX9^+^EdU^+^ progenitors (M), and SOX9^+^ cell distribution across zones (N) in the E17.5 neocortex. (O) Representative immunofluorescence images of EdU (red) and SOX9 (green) co‐staining in the neocortex of control and *Nsd2* ^Emx1−cKO^ mice. Pregnant dams received EdU injections at E16.5, and embryonic brains were collected at E18.5. (P) Representative immunofluorescence images of EdU (red) and BLBP (green) co‐staining in the neocortex of control and *Nsd2* ^Emx1−cKO^ mice. (Q) Quantification of total EdU^+^ cells. (R) Quantification of SOX9^+^ cells in the cortex. (S) Quantification of SOX9^+^EdU^+^ double‐positive cells in the cortex. (T) Percentage of SOX9^+^EdU^+^ cells among total EdU^+^ cells in each cortical region (VZ/SVZ, IZ, and CP). (U) Percentage of BLBP^+^EdU^+^ cells among total EdU^+^ cells in each cortical region (VZ/SVZ, IZ, and CP). For all analyses of E16.5–E18.5 sections, positive cells were quantified within a rectangular region of interest (ROI; 300 µm width × 600 µm height) (*n* = 3–4 biological replicates). Scale bar, 100 µm. VZ, ventricular zone; SVZ, subventricular zone; IZ, intermediate zone; CP, cortical plate. Unpaired Student's *t*‐test or Welch's *t*‐test was used as appropriate. *p < 0.05, **p < 0.01, ***p < 0.001, ****p < 0.0001. Data are presented as mean ± SEM.

Next, we examined EGFR, a well‐established marker of gliogenic competence onset [28, 29]. By E17.5, EGFR^+^ progenitors were markedly downregulated in both *Nsd2* ^Emx1−cKO^ and *Nsd2* ^hGFAP−cKO^ cortex, particularly within the ventricular/subventricular zone (VZ/SVZ) (Figure 2A–F). Concurrently, the gliogenic progenitor markers ALDH1L1, GFAP and SOX9 [27] were significantly reduced in the VZ/SVZ (Figure 2G–N), indicating a failure of RGCs to acquire gliogenic competence. Furthermore, a 2 h EdU pulse‐labeling experiment at E17.5 revealed a significant reduction in total proliferating (EdU^+^) cells within the VZ/SVZ, with a pronounced decrease specifically in proliferating astrocyte progenitors (SOX9^+^EdU^+^) (Figure 2L,M). Similar gliogenic defects were also observed in *Nsd2* ^hGFAP−cKO^ mice, as indicated by reduced GFAP and SOX9 expression (Figure S6). To further characterize the gliogenic potential of proliferating RGCs, we performed EdU lineage‐labeling experiments with region‐specific quantitative analyses. EdU was administered at E16.5 during the peak gliogenic stage, and embryonic brains were collected at E18.5 for co‐immunostaining with SOX9 or BLBP (Figure 2O,P). Quantification was performed separately in the VZ/SVZ, IZ, and CP. In *Nsd2* ^Emx1−cKO^ cortices, the total numbers of EdU^+^, SOX9^+^, and BLBP^+^ cells were all significantly decreased (Figure 2Q–S). Moreover, the percentages of SOX9^+^EdU^+^/EdU^+^ and BLBP^+^EdU^+^/EdU^+^ cells were significantly reduced in the VZ/SVZ, with similar reductions observed in the IZ and CP (Figure 2T,U). These findings indicate that NSD2 deficiency impairs gliogenic fate specification and proliferative expansion during the neurogenesis‐to‐gliogenesis transition, thereby reducing the production of gliogenic precursors and astrocytes.

To further characterize gliogenic intermediate progenitors, we performed OLIG2 and EGFR co‐immunostaining on E17.5 cortical sections. OLIG2^+^EGFR^+^ cells represent gliogenic intermediate progenitors generated during the neurogenesis‐to‐gliogenesis transition. Our analyses showed that OLIG2^+^EGFR^+^ double‐positive cells were significantly reduced in *Nsd2* ^Emx1−cKO^ cortices compared with controls (Figure S7A,C). Because gliogenesis includes both astrocyte and oligodendrocyte lineage development, we next examined whether NSD2 deficiency affects the oligodendrocyte lineage. Co‐immunostaining for SOX10 and OLIG2 at E17.5 showed significant reductions in SOX10^+^ cells, OLIG2^+^ cells, and SOX10^+^OLIG2^+^ double‐positive cells in *Nsd2* ^Emx1−cKO^ mice (Figure S7B,D–F), indicating impaired OPC specification or expansion. To assess whether these early deficits affect later myelination, we performed MBP immunostaining at P14 (Figure S7G), which showed qualitatively comparable MBP expression between control and cKO mice, suggesting that overall myelination is not markedly disrupted at this stage based on qualitative examination.

To determine whether these early gliogenic defects persist throughout development, we next analyzed the temporal dynamics of BLBP^+^ progenitors from embryonic to postnatal stages. Consistent with the lineage‐tracing results, BLBP^+^ cell numbers were significantly reduced in *Nsd2* ^Emx1−cKO^ mice at E16.5, E18.5, and P0 (Figure S8A–C). Unexpectedly, at later stages (P7‐P14), BLBP^+^ cell numbers in *Nsd2* ^Emx1−cKO^ cortices were significantly increased relative to controls (Figure S8A,D,E). Although these cells showed no obvious morphological abnormalities, their accumulation suggests that NSD2 deficiency alters astrocyte maturation, turnover, or lineage progression during postnatal development. Together, these findings suggest that NSD2 deficiency delays, rather than completely blocks, gliogenic progression during corticogenesis.

### 2.4 NSD2 Promotes Astrocytic Fate Through its Methyltransferase Activity

**FIGURE 3:**
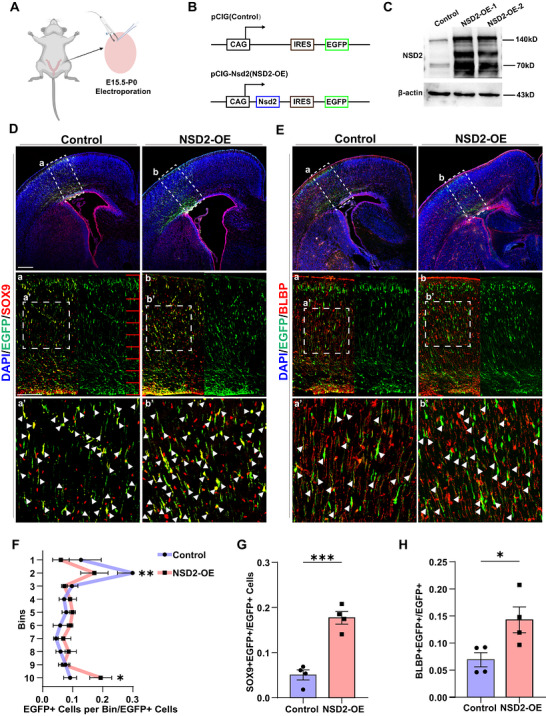
Overexpression of NSD2 promotes astrogenesis in the developing cortex. (A) Schematic diagram of the in utero electroporation (IUE) procedure. Plasmids were injected into the lateral ventricles of E15.5 embryos, and brains were harvested for analysis at P0. (B) Schematic representation of the constructs used: the control vector (pCIG) and the NSD2‐overexpressing vector (CAG‐NSD2‐IRES‐EGFP). (C) Western blot validation confirming the overexpression efficiency of NSD2 in HEK293T cells. (D, E) Representative immunofluorescence images of P0 cortical sections electroporated at E15.5. Sections were stained for EGFP (green) to label electroporated cells and the astrocyte markers SOX9 (red, D) or BLBP (red, E). (F) Quantification of the laminar distribution of EGFP^+^ cells. The cortex was divided into 10 equal bins from the ventricular surface to the pial surface (as indicated by the red bar in D), and the percentage of EGFP^+^ cells in each bin was calculated. (G, H) Quantification of astrogenic differentiation, shown as the percentage of EGFP^+^ cells co‐expressing SOX9 (G) or BLBP (H). Scale bar, 200 µm. Data are presented as mean ± SEM. *n* = 4 brains per group. *p < 0.05, **p < 0.01, ***p < 0.001, ****p < 0.0001; by two‐way ANOVA with Sidak's multiple comparisons test (F) or Unpaired Student's *t*‐test or Welch's *t*‐test (G, H).

We next asked whether NSD2 is sufficient to drive gliogenesis by performing in utero electroporation of an *Nsd2* overexpression construct at E15.5, followed by analysis at P0 (Figure 3A–C). Compared with controls, NSD2 overexpression altered the laminar distribution of transfected progeny, with fewer cells located in the upper cortical layers and more cells retained in periventricular regions (Figure 3D,F). Co‐immunostaining revealed that NSD2 overexpression significantly increased the proportion of transfected (EGFP^+^) cells co‐expressing the astrocyte markers SOX9 or BLBP, compared with controls (Figure 3D,E,G,H). These gain‐of‐function results complement the loss‐of‐function data, demonstrating that elevated NSD2 levels can promote astrogenic commitment.

To determine whether the astrogenic function of NSD2 depends on its methyltransferase activity, we generated a catalytically inactive NSD2 mutant carrying the Y1179A substitution within the SET domain (Figure S9A) [30]. Western blot analysis confirmed the expression of shNsd2, wild‐type *Nsd2*, and the catalytic mutant constructs (Figure S9B). Rescue experiments were then performed by co‐electroporating shNsd2 together with either wild‐type *Nsd2* or the catalytically inactive mutant at E15.5, followed by analysis at P0 (Figure S9C). While wild‐type *Nsd2* effectively rescued the reduction in BLBP^+^ astrocytic cells caused by *Nsd2* knockdown, the catalytic mutant failed to do so (Figure S9D). In addition, wild‐type *Nsd2*, but not the catalytic mutant, restored the distribution of co‐transfected cells within the cortical plate and reduced their abnormal retention in the VZ/SVZ (Figure S9E,F). These findings indicate that the astrogenic function of NSD2 requires its histone methyltransferase activity.

### 2.5 *Nsd2* Deletion Impairs H3K36me2 Deposition and Attenuates MAPK/ERK Signaling

**FIGURE 4:**
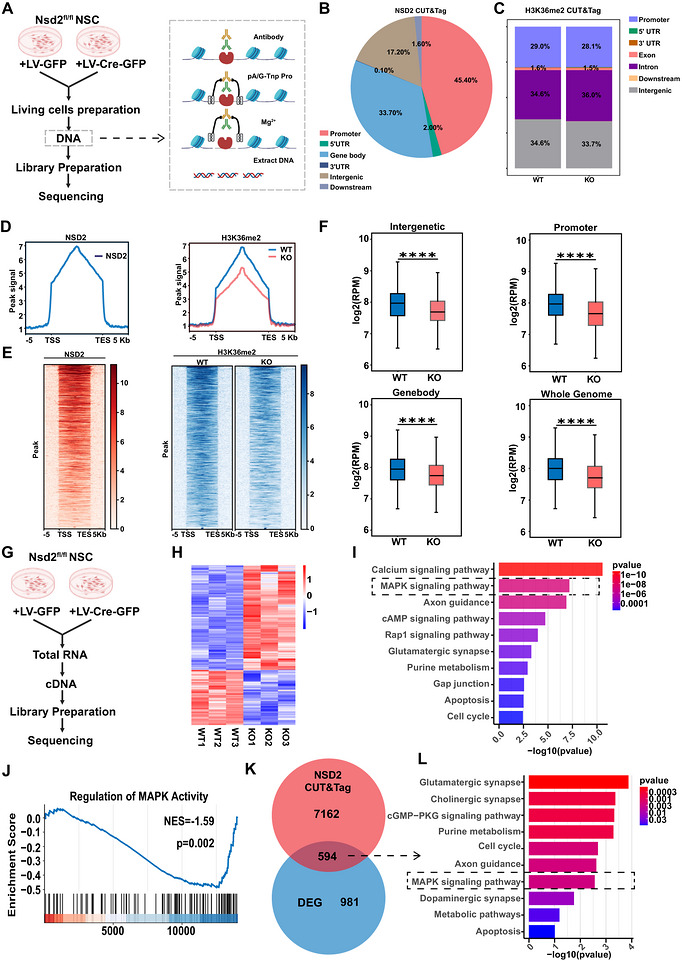
Loss of NSD2 results in a global reduction of H3K36me2 and attenuation of MAPK pathway activity. (A) Schematic illustration of the CUT&Tag workflow performed in embryonic neural stem cells (NSCs). (B, C) Genome‐wide distribution of NSD2 (B) and H3K36me2 (C) signals across annotated genomic features. (D) Metagene profiles showing average enrichment of NSD2 and H3K36me2 across transcription start site (TSS) to transcription end site (TES) regions in E15.5 NSCs. (E) Heatmap showing NSD2 and H3K36me2 signal intensity centered on the genomic regions shown in (D). (F) Quantification of normalized H3K36me2 CUT&Tag reads in wild‐type (WT, blue) and NSD2 knockout (KO, red) E15.5 NSCs across intergenic regions, promoters (±3 kb from the TSS), gene bodies, and genome‐wide regions. (G) Schematic overview of the RNA‐seq experimental workflow. (H) Hierarchical clustering of differentially expressed genes (DEGs) between WT and KO NSCs at E17.5. (I) KEGG pathway enrichment analysis of DEGs identified in WT and KO NSCs at E17.5. (J) Gene Set Enrichment Analysis (GSEA) plot showing significant downregulation of genes involved in the regulation of MAPK activity in E17.5 NSD2 KO NSCs. (K) Venn diagram showing the overlap between DEGs identified by RNA‐seq and NSD2 direct target genes identified by CUT&Tag. (L) KEGG pathway enrichment analysis of the overlapping genes shown in (K). CUT&Tag and RNA‐seq experiments were performed with three independent biological replicates per group. Statistical significance: *p < 0.05, **p < 0.01, ***p < 0.001, ****p < 0.0001; by Wilcoxon rank‐sum test (F).

Given that the astrogenic function of NSD2 depends on its methyltransferase activity, we next sought to define the downstream molecular mechanisms underlying NSD2‐mediated gliogenesis. We performed integrated CUT&Tag and RNA‐seq analyses in control and *Nsd2*‐deficient neural stem cells (NSCs) to determine how H3K36me2 depletion affects transcriptional programs (Figure 4A,G). NSD2 occupancy was predominantly enriched at promoters and gene bodies, with a smaller fraction detected in intergenic regions (Figure 4B). *Nsd2* deletion in NSCs using Cre‐expressing lentivirus led to a genome‐wide reduction of H3K36me2 occupancy, particularly at promoters and across transcription start site (TSS) to transcription end site (TES) regions where NSD2 is enriched, consistent with its enzymatic function (Figure 4C–F).

Transcriptomic profiling revealed more upregulated than downregulated genes in *Nsd2* KO NSCs (Figure 4H). KEGG pathway and GSEA analyses identified a marked downregulation of MAPK/ERK signaling in *Nsd2* KO NSCs (Figure 4I,J), consistent with the observed defects in gliogenesis. Intersection of NSD2 CUT&Tag targets with differentially expressed genes (DEGs) yielded 594 overlapping genes (Figure 4K), which were also enriched for MAPK/ERK signaling components (Figure 4L). To assess the in vivo relevance of our findings, we performed RNA‐seq on E14.5 *Nsd2* ^Emx1−cKO^ cortical tissues and found that both upregulated and downregulated genes shared between the cKO cortex and E17.5 *Nsd2*‐deficient NSCs were enriched in development‐related processes. Notably, the overlapping downregulated genes indicated consistent alterations in MAPK/ERK signaling (Figure S10A–F). To validate this in vivo, we performed p‐ERK immunofluorescence on E17.5 cortical sections (Figure S10G). Quantification revealed a significant reduction in p‐ERK^+^ cell density in the cKO cortex (Figure S10H), confirming that NSD2 deficiency suppresses ERK phosphorylation during cortical development. Collectively, these results demonstrate that NSD2‐dependent H3K36me2 is required to maintain MAPK/ERK signaling.

### 2.6 NSD2 Directly Binds and Activates the *Egfr* Promoter

**FIGURE 5:**
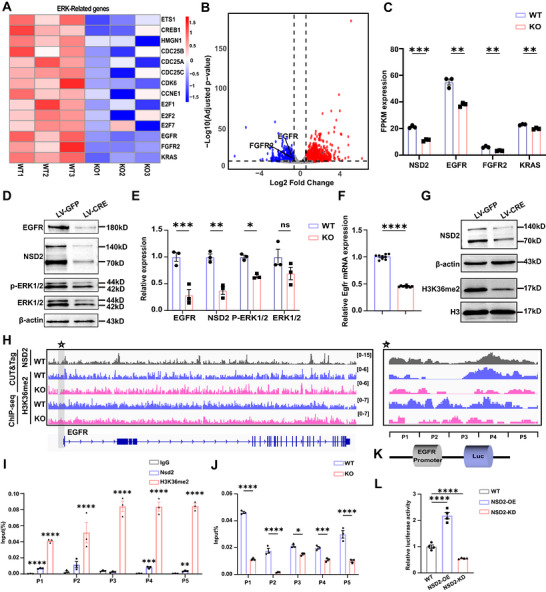
NSD2 directly binds the *Egfr* promoter and regulates its expression. (A) Heatmap depicting the relative expression of ERK signaling–related genes derived from RNA‐seq data. (B) Volcano plot showing differentially expressed genes in *Nsd2* KO NSCs. (C) Bar graph showing the FPKM values of selected ERK signaling–related genes in NSCs. (D, E) Western blot analysis (D) and quantification (E) of indicated protein levels in *Nsd2* KO NSCs. (F) qPCR analysis of *Egfr* mRNA levels in *Nsd2* KO NSCs. (G) Western blot analysis of global H3K36me2 levels in *Nsd2* KO NSCs. (H) IGV tracks displaying NSD2 and H3K36me2 occupancy at the *Egfr* locus (left). The ChIP‐seq data shown in this figure were obtained from the published study by Kinoshita et al. [31]. A magnified view of the 2‐kb promoter region is shown on the right, highlighting the five specific DNA fragments selected for cloning. (I) ChIP–qPCR analysis of NSD2 binding and H3K36me2 enrichment at the *Egfr* promoter. (J) ChIP–qPCR analysis quantifying H3K36me2 levels at the *Egfr* promoter in *Nsd2* KO NSCs. (K) Schematic illustration of the dual‐luciferase reporter constructs. (L) Dual‐luciferase reporter assays assessing *Egfr* promoter activity following *Nsd2* knockdown and overexpression. Data are presented as mean ± SEM from three independent biological replicates. Statistical significance was determined using an Unpaired Student's *t*‐test or Welch's *t*‐test (F), multiple unpaired *t*‐tests (I, L), two‐way ANOVA (J). *p < 0.05, **p < 0.01, ***p < 0.001, ****p < 0.0001.

Re‐analysis of NSC RNA‐seq data revealed coordinated suppression of ERK‐MAPK pathway genes in *Nsd2*‐deficient NSCs (Figure 5A), including both downstream effectors (e.g., *Ets1*, *Creb1*) and upstream regulators such as receptor tyrosine kinases (e.g., *Fgfr2*, *Egfr*) and the RAS family member *Kras*. Volcano plot analysis further identified *Fgfr2* and *Egfr* among the most significantly downregulated genes in *Nsd2*‐deficient NSCs (Figure 5B). Consistently, RNA‐seq expression analyses showed reduced levels of *Nsd2*, *Egfr*, *Fgfr2*, and *Kras* in KO NSCs relative to WT controls (Figure 5C). Given the established role of EGFR in gliogenesis, we next focused on validating its dysregulation. Western blot revealed that loss of NSD2 resulted in a significant reduction in the protein levels of EGFR and its downstream effector phosphorylated ERK (p‐ERK) (Figure 5D,E). Consistently, qPCR analysis showed a significant reduction in *Egfr* mRNA levels in *Nsd2* KO NSCs (Figure 5F), indicating that NSD2 regulates *Egfr* expression at the transcriptional level. In parallel, H3K36me2 levels were globally decreased in *Nsd2* KO NSCs (Figure 5G), consistent with the role of NSD2 as a major H3K36me2 methyltransferase in NSCs.

IGV visualization and CUT&Tag profiling revealed co‐localization of NSD2 binding and H3K36me2 enrichment at the *Egfr* locus [31], particularly within the promoter region (Figure 5H). To further examine this regulatory region, we designed five primer sets spanning the *Egfr* promoter (Figure 5H). ChIP–qPCR analysis confirmed enrichment of both NSD2 and H3K36me2 at the *Egfr* promoter in control NSCs, with the strongest signals detected at sites P1, P4, and P5 (Figure 5I). Importantly, *Nsd2* knockout markedly reduced H3K36me2 enrichment at these promoter regions (Figure 5J). To determine whether NSD2 directly regulates *Egfr* transcription, we generated dual‐luciferase reporter constructs containing the *Egfr* promoter sequence (Figure 5K). Dual‐luciferase assays showed that NSD2 knockdown significantly suppressed *Egfr* promoter activity, whereas NSD2 overexpression enhanced it (Figure 5L), demonstrating direct transcriptional activation of *Egfr* by NSD2. Collectively, these results establish a mechanism whereby NSD2‐mediated H3K36me2 deposition promotes *Egfr* transcription and downstream ERK‐MAPK signaling in NSCs.

### 2.7 Pharmacological Activation of p‐ERK Rescues Astrogenic Defects by *Nsd2* Loss

**FIGURE 6:**
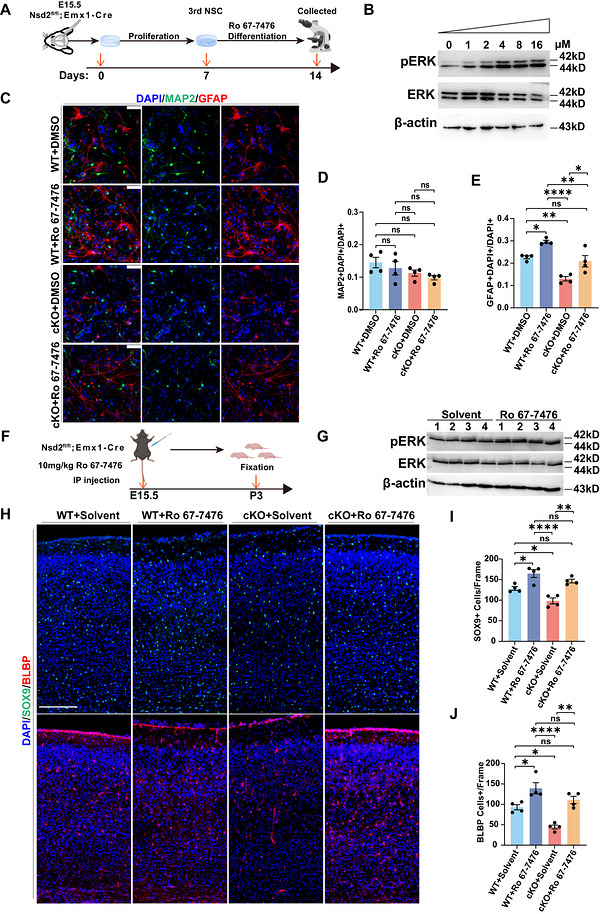
Pharmacological activation of p‐ERK rescues astrocyte generation deficits caused by Nsd2 deletion. (A) Schematic of the in vitro rescue experiment. NSCs were isolated from E15.5 *Nsd2* ^fl/fl^;Emx1‐Cre mice, cultured for 7 days (passage 3), and treated with Ro 67–7476 during differentiation. Samples were collected after 7 days. (B) Dose‐response analysis of p‐ERK expression in NSCs treated with the indicated concentrations of Ro 67–7476 (0, 1, 2, 4, 8, 16 µM). (C) Representative immunofluorescence images of differentiated NSCs stained for MAP2 (neurons) and GFAP (astrocytes) following vehicle or Ro 67–7476 treatment. Scale bar, 100 µm. (D, E) Quantification of the proportions of MAP2^+^ (D) and GFAP^+^ (E) cells from the experiment in (C). Data were analyzed using one‐way ANOVA. (F) Schematic of the in vivo rescue experiment. Pregnant mice (E15.5) received a single intraperitoneal injection of Ro 67–7476 (10 mg kg^−1^), and cortices were analyzed at P3. (G) Western blot analysis of p‐ERK expression in cortices of vehicle‐ and Ro 67‐7476‐treated mice. (H) Representative immunofluorescence images of P3 cortices stained for astrocyte markers SOX9 and BLBP. Scale bar, 200 µm. (I, J) Quantification of the density of SOX9^+^ (I) and BLBP^+^ (J) cells from the experiment in (H), statistical significance was determined by one‐way ANOVA. Data are presented as mean ± SEM; *n* = 4 biological replicates per group. *p < 0.05, **p < 0.01, ***p < 0.001, ****p < 0.0001.

We next asked whether impaired ERK signaling contributes to the astrogenic defects caused by *Nsd2* loss. NSCs isolated from *Nsd2* ^Emx1−cKO^ mice were treated with the p‐ERK activator Ro 67–7476 throughout a 7‐day differentiation period, followed by immunostaining for the neuronal marker MAP2 and the astrocytic marker GFAP (Figure 6A). The efficacy of Ro 67–7476 was first evaluated across multiple concentrations, and 4 µM was selected for subsequent experiments (Figure 6B). Consistent with our previous findings, *Nsd2* deficiency impaired astrocyte differentiation while largely sparing neuronal differentiation (Figure 6C–E). Importantly, treatment with Ro 67–7476 (4 µM) restored p‐ERK levels and significantly rescued astrocyte generation without significantly affecting neuronal differentiation (Figure 6C–E). These results are consistent with our in vivo observations of reduced astrocyte numbers upon *Nsd2* deletion and indicate that the astrogenic deficit is dependent on p‐ERK activity.

This rescue was validated in vivo. Ro 67–7476 was administered to pregnant mice by intraperitoneal injection at E15.5, and cortical tissues from the offspring were collected at P3 for analysis (Figure 6F). To verify effective pathway activation in vivo, *p*‐ERK levels were examined in the offspring cortices. Ro 67–7476 treatment increased p‐ERK levels relative to vehicle‐treated controls, as confirmed by both western blot and immunofluorescence analyses (Figure 6G and Figure S11A,B). This pharmacological intervention significantly increased the density of SOX9^+^ and BLBP^+^ astrocytes in *Nsd2* ^Emx1−cKO^ cortices, while neuronal markers remained unchanged (Figure 6H–J and Figure S11C,D). These findings indicate that impaired ERK signaling is a major contributor to the astrogenic defects caused by *Nsd2* deficiency and that these defects are pharmacologically reversible.

### 2.8 *Nsd2* Deficiency Causes Cognitive and Behavioral Deficits

Given that intellectual disability is a feature of Wolf‐Hirschhorn syndrome (WHS), and that *NSD2* is one of the key causative genes within the WHS critical region [32, 33], we performed a battery of behavioral tests in *Nsd2* ^Emx1−cKO^ mice to determine whether severe cortical *Nsd2* loss leads to cognitive and behavioral abnormalities.

**FIGURE 7:**
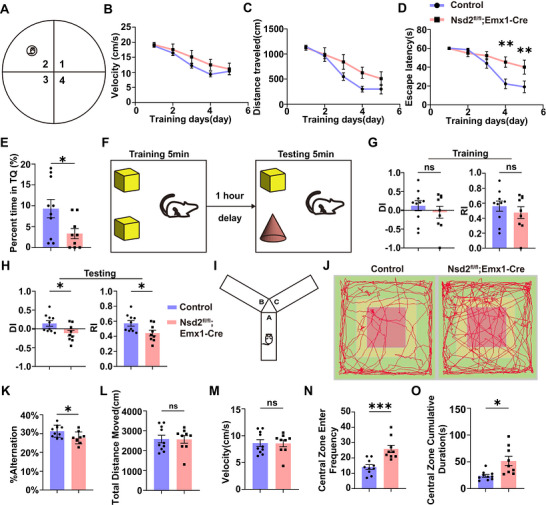
*Nsd2* deficiency impairs learning and memory behaviors. (A) Schematic diagram of the Morris water maze (MWM) test; the escape platform was located in the second quadrant. (B–D) Quantification of swimming velocity (B), total distance traveled (C), and escape latency (D) during the training days. Data were analyzed using two‐way ANOVA followed by Sidak's multiple comparisons test. (E) Quantification of the time spent in the target quadrant during the probe test. Data were analyzed using a Welch's *t*‐test. (F) Schematic diagram of the novel object recognition (NOR) test. The yellow cube represents the familiar object, and the red cone represents the novel object. (G, H) Quantification of the discrimination index (DI) (G) and recognition index (RI) (H) before and after training. Data were analyzed using an unpaired Student's *t*‐test. (I) Schematic diagram of the Y‐maze test; mice started from arm A. (J) Representative movement tracks in the open field test (OFT). (K) Quantification of spontaneous alternation percentage in the Y‐maze. Data were analyzed using an unpaired Student's *t*‐test. (L–O) Quantification of total distance traveled (L), velocity (M), number of center crossings (N), and time spent in the center (O) in the open field test. Data were analyzed using an unpaired Student's *t*‐test or Welch's *t*‐test. Data are presented as mean ± SEM. *n* = 11 (WT) and *n* = 9 (cKO) mice per group. *p < 0.05, **p < 0.01, ***p < 0.001, ****p < 0.0001.

To explore a potential association between *Nsd2* loss and cognitive function, human orthologs of DEGs from the E14.5 RNA‐seq dataset were cross‐referenced with genes listed in the OMIM database associated with intellectual disability. This analysis identified 18 overlapping genes (Figure S12A,B), suggesting a potential link between *Nsd2* deficiency and neurodevelopmental disorders involving cognitive impairment in mice. To assess long‐term spatial learning and memory, *Nsd2* ^Emx1−cKO^ mice were tested in the Morris water maze (MWM) using a 5‐day training protocol followed by a probe test on day 6. During the early training sessions, performance was comparable between cKO and control mice. By day 4, control mice efficiently located the platform, whereas *Nsd2* ^Emx1−cKO^ mice required longer escape latencies. This impairment persisted in the probe test, indicating impaired spatial learning and memory in *Nsd2*‐deficient mice (Figure 7A–E). The novel object recognition (NOR) test was performed to assess recognition memory using the discrimination index (DI) and recognition index (RI). *Nsd2* ^Emx1−cKO^ mice showed a lower preference for the novel object compared with controls, resulting in decreased RI and DI scores, consistent with impaired recognition memory (Figure 7F–H). Similarly, the Y‐maze test revealed a reduced percentage of spontaneous alternations in *Nsd2* ^Emx1−cKO^ mice, indicating deficits in working memory (Figure 7I,K). In the open field test (OFT), locomotor activity was comparable between *Nsd2* ^Emx1−cKO^ and control mice. *Nsd2* ^Emx1−cKO^ mice, however, showed an increased frequency of entries into the center zone, suggesting reduced anxiety‐like behavior (Figure 7J, L–O). These findings indicate that *Nsd2* ^Emx1−cKO^ mice exhibit impairments in learning and memory, consistent with cognitive dysfunction.

## 3 Discussion

Our study establishes NSD2 as a master epigenetic regulator essential for cortical gliogenesis (Figure S13). We uncover an NSD2‐H3K36me2‐EGFR‐ERK signaling axis that drives the fate transition of radial glial cells from neurogenesis to gliogenesis (Figure S13). This work provides mechanistic insight into how NSD2‐dependent regulation of glial lineage development may contribute to neurodevelopmental abnormalities associated with Wolf‐Hirschhorn syndrome and related disorders.

### 3.1 NSD2: A Rate‐Limiting Regulator of the Epigenetic Control of Gliogenesis

The NSD family, comprising NSD1, NSD2, and NSD3, consists of H3K36me2‐specific histone methyltransferases that exhibit distinct temporal functions during cortical development [34]. While NSD1 is crucial for neuronal lineage integrity during peak neurogenesis [18, 35], our data reveal that NSD2 functions later, during late corticogenesis, to promote gliogenic competence. This conclusion is supported by temporal expression profiling showing that NSD2 expression increased markedly during the onset and progression of gliogenesis (Figure S1B), the selective impairment of gliogenic progression following *Nsd2* loss, and the inability of the catalytically inactive NSD2‐Y1179A mutant to rescue astrogenic defects (Figure 2 and Figures S5, S6, and S9). Consistent with this interpretation, loss of NSD2 impaired the acquisition of gliogenic identity, as reflected by reduced expression of gliogenic markers (ALDH1L1, GFAP, SOX9, EGFR) at E17.5, whereas early neurogenic stages (E12.5‐E14.5) were largely unaffected (Figure 2 and Figures S5 and S6). Together, these findings support a model in which distinct H3K36 methyltransferases act sequentially during cortical development to coordinate stage‐specific lineage transitions. In this model, NSD1 promotes neuronal lineage progression during neurogenesis, whereas NSD2 is required for the proper timing and execution of the transition toward gliogenesis.

Notably, the partial rather than complete disruption of gliogenic progression observed in NSD2‐deficient mice—with a subset of RGCs retaining gliogenic potential—suggests that NSD2 functions as a rate‐limiting regulator rather than an all‐or‐none determinant of gliogenesis. This interpretation is consistent with the observation that the proportions of SOX9^+^EdU^+^/EdU^+^ and BLBP^+^EdU^+^/EdU^+^ cells were significantly reduced but not abolished in the mutant cortex (Figure 2T,U). These findings suggest that at least a subset of radial glial cells remains competent for gliogenic differentiation in the absence of NSD2, possibly reflecting heterogeneity among radial glial subpopulations in their responsiveness to gliogenic cues, including EGFR dosage‐dependent responsiveness [28]. Moreover, NSD2 deficiency also affected the oligodendrocyte lineage, as numbers of SOX10^+^, OLIG2^+^, and SOX10^+^OLIG2^+^ cells were significantly reduced at E17.5 (Figure S7), suggesting that NSD2 broadly contributes to late‐stage glial lineage competence.

The dynamic postnatal changes in BLBP expression further suggest that NSD2 regulates not only gliogenic initiation but also the temporal progression of astroglial development. While BLBP expression was markedly reduced during embryonic and early postnatal stages (E16.5‐P3), BLBP‐positive cells accumulated at later postnatal stages in the mutant cortex (Figure S8). Although these cells did not display obvious morphological abnormalities, their accumulation suggests disrupted temporal control of astrocyte maturation and lineage progression during postnatal development. To explain this late‐stage increase, we propose two non‐mutually exclusive possibilities. First, compensatory activation of other gliogenic signaling pathways (e.g., JAK‐STAT, CNTF/LIF) during later stages may partially offset the reduction in ERK signaling. Second, early gliogenic defects and/or increased apoptosis may induce compensatory astroglial responses, leading to secondary accumulation of BLBP‐positive cells during postnatal development. Of note, while oligodendrocyte precursor cells (OPCs) were reduced at embryonic stages, postnatal myelination appeared largely preserved based on qualitative MBP staining at P14, suggesting that surviving OPCs may undergo compensatory expansion (Figure S7).

We propose that NSD2 primes radial glial cells for gliogenic competence by establishing a permissive epigenetic landscape through H3K36me2 deposition, thereby enabling the timely execution of glial lineage transitions during corticogenesis.

### 3.2 A Direct Epigenetic‐Signaling Axis to Control the Switch

Mechanistically, we found that NSD2 maintains an active chromatin state at the *Egfr* promoter, a key upstream regulator of the MAPK‐ERK signaling pathway. MAPK‐ERK signaling has been previously shown to promote astrocyte production [36, 37, 38]. By depositing H3K36me2 at the promoter, NSD2 ensures robust EGFR expression, which in turn drives the downstream ERK signaling essential for gliogenic commitment. Notably, the regulation of the ERK pathway by NSD2 has been reported in other biological contexts, including cancer, highlighting a broader role of this regulatory relationship [39]. This direct link from a histone modifier to a key receptor tyrosine kinase pathway illustrates how epigenetic regulators can serve as nodal points, coupling chromatin states to signaling programs that influence cell fate decisions. The rescue of astrogenesis by pharmacological ERK activation, both in differentiating NSCs in vitro and in the embryonic brain in vivo, effectively underscores that attenuated ERK signaling represents the critical downstream event underlying the astrogenic failure observed in *Nsd2*‐deficient mice. This study thus establishes an “NSD2–H3K36me2–EGFR–ERK” axis, elucidating a precise mechanism by which NSD2 epigenetically sustains the expression of a core growth factor receptor, providing the requisite signaling drive for the gliogenic fate transition.

### 3.3 Linking Glial Development to Cognitive Function

Our findings bridge molecular, cellular, and behavioral levels to provide insight into the potential contribution of NSD2‐dependent glial development to cognitive function. *Nsd2* ^Emx1−cKO^ mice exhibit growth retardation, microcephaly, and cognitive deficits, features that overlap with aspects of neurodevelopmental disorders associated with NSD2 haploinsufficiency, including Wolf‐Hirschhorn syndrome and Rauch‐Steindl syndrome. We demonstrate that cognitive deficits in *Nsd2* ^Emx1−cKO^ mice are accompanied by a marked loss of cortical astrocytes. In addition, our analyses revealed that oligodendrocyte precursor cells (OPCs) are also significantly reduced in *Nsd2*‐deficient cortices at E17.5, as evidenced by decreased numbers of SOX10^+^, OLIG2^+^, and SOX10^+^OLIG2^+^ double‐positive cells (Figure S7). Thus, *Nsd2* deficiency broadly impairs gliogenesis, affecting both astrocyte and oligodendrocyte lineages. Both astrocyte and OPC/oligodendrocyte dysfunction have been increasingly implicated in cognitive impairment and neurodevelopmental disorders [40, 41, 42, 43, 44]. Accordingly, impaired gliogenesis caused by NSD2 deficiency may disrupt cortical circuit formation and function, thereby contributing to the observed behavioral deficits. Collectively, these findings suggest that defective glial development represents a potentially important and previously underappreciated cellular mechanism in NSD2‐associated neurodevelopmental phenotypes. Future studies using heterozygous *Nsd2* knockout models will be essential to determine whether the gliogenic defects observed here are recapitulated under more physiologically relevant conditions.

## 4 Limitations and Future Directions

Despite establishing NSD2 as a key regulator of the gliogenic switch, several questions remain. First, this study utilized conditional homozygous *Nsd2* deletion models, whereas patients with NSD2‐associated disorders, including Wolf‐Hirschhorn syndrome and Rauch‐Steindl syndrome, typically carry heterozygous loss‐of‐function mutations (haploinsufficiency). Therefore, caution is warranted when extrapolating our findings to human disease. Future studies using heterozygous *Nsd2* knockout models will be important to determine which phenotypes are preserved under more physiologically relevant conditions. Second, given the pronounced spatiotemporal heterogeneity of RGCs, single‐cell multi‐omics will identify RGC subpopulations most vulnerable to NSD2 deficiency. Third, although *Egfr* emerges as a central downstream target, NSD2 likely orchestrates a broader molecular program, potentially involving other pathways to ensure the fidelity of gliogenic commitment. Fourth, the potential crosstalk between H3K36me2 and other epigenetic marks warrants further investigation to determine whether their redistribution contributes to transcriptional repression following NSD2 loss. Finally, the modest reduction of upper‐layer neurons raises the question of whether the observed behavioral deficits arise from glial loss, neuronal dysfunction, or their synergistic interaction, potentially through non–cell‐autonomous mechanisms. Decoupling the contributions of glial loss and mild neuronal dysfunction to the behavioral phenotype will be crucial to fully elucidate the cellular basis of NSD2‐associated neurodevelopmental phenotypes.

## 5 Conclusions

In summary, we identify NSD2 as a key epigenetic regulator of the gliogenic switch through modulation of the EGFR‐ERK signaling pathway. This work advances our understanding of how histone methylation guides brain development and provides a mechanistic framework for exploring how NSD2‐dependent chromatin regulation may be involved in neurodevelopmental phenotypes associated with NSD2 dysfunction, including intellectual disability. These findings also highlight potential pathways that may be explored for therapeutic intervention.

## 6 Methods

### 6.1 Mice

Mice were maintained on a C57BL/6J genetic background and housed in a specific pathogen‐free (SPF) facility under a 12 h light/dark cycle at a controlled temperature (20–24°C), with ad libitum access to food and water. All efforts were made to minimize animal suffering and to reduce the total number of animals used. For all experiments except behavioral tests, 3–4 mice per group were randomly selected; for behavioral assays, 9–10 mice per group were used. Two conditional knockout (cKO) mouse models were established. *Nsd2* ^fl/fl^ mice (Viewsolid Biotech) were crossed with *Emx1^IRES^‐*Cre mice (provided by Dr. Zhengang Yang, Fudan University; JAX stock #005628) or *hGFAP‐*Cre mice (provided by Dr. Weimin Zhong, Yale University). The initial cross yielded *Nsd2* ^fl/+^; *Emx1‐*Cre and *Nsd2* ^fl/+^; *hGFAP‐*Cre male mice, which were subsequently crossed with *Nsd2* ^fl/fl^ females to generate experimental cohorts: *Nsd2* ^fl/fl^; *Emx1‐*Cre (designated as *Nsd2* ^Emx1−cKO^) and *Nsd2* ^fl/fl^; *hGFAP‐*Cre (designated as *Nsd2* ^hGFAP−cKO^). *Nsd2* ^fl/fl^ littermates lacking the Cre transgene served as controls. For IUE experiments, pregnant ICR mice were obtained from the Laboratory Animal Center of Peking University Health Science Center. Both male and female mice were included in all analyses, and no significant sex‐related differences were observed.

### 6.2 Plasmid Construction

To knockdown *Nsd2* expression, short hairpin RNA (shRNA) sequences targeting mouse *Nsd2* were cloned into the pLL3.7 vector. For overexpression and rescue experiments, the full‐length coding sequences of *Nsd2* and *Egfr* were amplified and subcloned into the pCIG expression vector. To evaluate transcriptional regulation, the *Egfr* promoter region was PCR‐amplified and inserted into the pGL3‐Basic luciferase reporter vector. All plasmids were purified using QIAGEN Plasmid Mini or Maxi kits according to the manufacturer's protocols.

### 6.3 Cell Culture

*Primary NSC Culture*: Pregnant mice were anesthetized via intraperitoneal injection of 1.25% Avertin (2,2,2‐Tribromoethanol for ready use, Nanjing Aibei Biotechnology, China) at a dose of 0.2 mL/10 g body weight, followed by euthanasia via cervical dislocation. After immersion in 75% ethanol for 5 min, embryos were surgically harvested under sterile conditions. The cerebral cortices were then microdissected and rinsed thoroughly with ice‐cold normal saline (0.9% NaCl) to eliminate residual blood and meninges. The isolated cortical tissues were pre‐dissociated by gentle trituration using a P1000 pipette, followed by enzymatic digestion with ACCUTASE (Sigma, A6964) at 37°C for 5 min. The tissue fragments were then further triturated to achieve a single‐cell suspension. To ensure a uniform cell population, the suspension was passed through a 40‐µm cell strainer (Falcon, 352340) to remove cell aggregates and subsequently centrifuged at 800 rpm for 5 min to pellet the cells. The harvested cells were resuspended and seeded at a density of 1 × 10^6^ cells mL^−1^ in 10‐cm non‐adherent culture dishes. Cells were maintained in complete NSC proliferation medium composed of serum‐free DMEM/F12 (Gibco, 12634028) supplemented with 1% GlutaMAX (Gibco, 35050061), 2% B‐27 Supplement (Thermo Fisher, 17504044), 20 ng mL^−1^ epidermal growth factor (EGF; PeproTech, AF‐100‐15‐100UG), 20 ng mL^−1^ basic fibroblast growth factor (bFGF; PeproTech, 100–18B‐100UG), and 1% penicillin‐streptomycin (Cell Resource Center, Institute of Basic Medical Sciences, CAMS/PUMC, Beijing, China). Cultures were maintained at 37°C in a humidified incubator with 5% CO_2_, with fresh EGF and bFGF supplemented every other day. To evaluate differentiation potential, dissociated single cells were plated onto glass coverslips (Biosharp, ya‐350) coated with Matrigel (Yeasen, 40182ES08). Cells were cultured in growth factor–free medium (DMEM/F12 supplemented with 1% GlutaMAX and 2% B‐27) for 7 days. The medium was replenished every 2–3 days, after which cells were fixed for immunofluorescence analysis.

*Cell Line Culture*: HEK‐293T and N1E‐115 cell lines were obtained from Cell Resource Center. Cells were maintained in high‐glucose DMEM (Gibco, C11995500BT) supplemented with 10% fetal bovine serum (FBS; Gibco, 10270106) and 1% penicillin‐streptomycin. Cultures were kept at 37°C in a humidified incubator with 5% CO_2_.

### 6.4 Cell Transfection and Drug Treatment

NSCs isolated from the cerebral cortices of *Nsd2* ^fl/fl^ embryos were plated onto Matrigel‐coated dishes 24 h prior to transduction. To induce *Nsd2* deletion, cells were infected with either Cre‐expressing lentivirus (GM‐0220LV24‐1; Genomeditech, Shanghai, China) or an empty vector control, both at a titer of 1 × 10^8^ TU mL^−1^. Cells were harvested for subsequent analyses 3–4 days post‐infection.

To pharmacologically activate ERK signaling, primary NSCs were treated with the ERK activator Ro 67–7476 (MedChemExpress, HY‐100403) at a final concentration of 4 µM. Control cells received an equivalent volume of DMSO as vehicle.

Plasmid transfections were performed using Lipofectamine 3000 (Thermo Fisher, L3000015) following the manufacturer's instructions. Briefly, adherent cells were seeded at appropriate densities prior to transfection. Lipofectamine 3000 was diluted in Opti‐MEM Reduced Serum Medium (Cell Resource Center, IBMS, CAMS), and 2.5 µg of plasmid DNA was mixed with P3000 Reagent in an equal volume of Opti‐MEM. The diluted DNA solution was then combined with the diluted Lipofectamine 3000 at a 1:1 ratio and incubated at room temperature for 15 min to allow complex formation. The DNA‐lipid complexes were added dropwise to the cells, which were subsequently incubated at 37°C for 2–4 days before downstream analyses.

### 6.5 Western Blot

Cells or tissue samples were harvested and lysed in ice‐cold TNTE lysis buffer supplemented with a protease inhibitor cocktail (PMSF, leupeptin, aprotinin, and pepstatin A). Lysates were incubated on ice for 30 min and centrifuged at 12 000 g for 30 min at 4°C. The supernatant was collected, and protein concentrations were determined using the BCA Protein Assay Kit (Thermo Fisher, 23227). Protein samples were denatured by boiling in loading buffer, separated by 10%–12% SDS‐PAGE, and transferred onto nitrocellulose membranes (Cytiva, 10600001) using a semi‐dry transfer system. Membranes were blocked with 5% non‐fat milk in TBST and incubated with primary antibodies (listed in Table S1) overnight at 4°C. Following TBST washes, membranes were incubated with HRP‐conjugated secondary antibodies for 2 h at room temperature. Immunoreactive bands were visualized using a chemiluminescence substrate (NCM Biotech, P10300B) and imaged with a digital system. Band intensities were quantified using ImageJ software and normalized to β‐actin. Data represent results from at least three independent experiments.

### 6.6 Dual‐Luciferase Reporter Assay

HEK‐293T cells were seeded into 24‐well plates and co‐transfected with the *Egfr* promoter‐luciferase reporter plasmid (pGL3‐Basic), the *Renilla* luciferase internal control vector (pRL‐TK), and the indicated effector plasmids. Cells were harvested 72 h post‐transfection. Firefly and *Renilla* luciferase activities were measured sequentially in the same sample using the Dual‐Luciferase Reporter Assay System (Promega, E1910) according to the manufacturer's instructions. Firefly luciferase activity was normalized to *Renilla* luciferase activity to account for transfection efficiency. Data are presented as mean ± SEM from three independent experiments, each performed in quadruplicate.

### 6.7 In Utero Electroporation

Pregnant mice at embryonic day 15.5 (E15.5) were anesthetized via intraperitoneal injection of Avertin at 0.2 mL/10 g body weight. The uterine horns were surgically exposed, and plasmid DNA mixtures were microinjected into the lateral ventricles of the embryos using a glass micropipette. Each mixture contained pCIG‐*Nsd2* or pCIG control vector (2.5 mg mL^−1^) combined with pCAG (0.5 mg mL^−1^). Electroporation was performed by applying five square‐wave pulses (50 V, 50 ms duration, 950 ms interval) across the uterine wall using tweezer electrodes connected to an electroporator. Following the procedure, the uterus was returned to the abdominal cavity, the abdominal wall was sutured, and pregnant mice were maintained on a heating pad until recovery.

### 6.8 Tissue Preparation

For embryonic and postnatal day 0 (P0) mice, brains were dissected in ice‐cold PBS. P0 pups were anesthetized and euthanized as previously described prior to dissection. The collected brains were immersion‐fixed in 4% paraformaldehyde (PFA; Sigma) at 4°C for 2 days. Postnatal day 3 (P3) mice were anesthetized and transcardially perfused with saline followed by 4% PFA. Brains were harvested and post‐fixed in 4% PFA at 4°C for 2 days. Following fixation, tissues were dehydrated in 25% sucrose at 4°C for 1–2 days until fully equilibrated. Brains were embedded in OCT compound (Zhongshan Golden Bridge, ZLI‐9302) and stored at −80°C. Serial frozen coronal sections (14–16 µm thick) were obtained using a cryostat (Leica, CM1900).

### 6.9 EdU Labeling, TUNEL Assay, and Immunofluorescence

To assess cell proliferation and apoptosis, EdU and TUNEL assays were performed. For proliferation analysis, pregnant mice at the indicated gestational stages were intraperitoneally injected with EdU (50 mg kg^−1^ body weight). Frozen brain sections were prepared as described above, and EdU incorporation was detected using the Click‐iT Plus EdU Alexa Fluor Imaging Kit (Thermo Fisher, C10337) according to the manufacturer's instructions. Apoptosis was evaluated on adjacent sections via TUNEL staining using the TUNEL Apoptosis Detection Kit (YSFluor 640; Yeasen Biotech) following the standard protocol.

For tissue immunofluorescence, cryosections were mounted onto positively charged adhesion slides (Zhongshan Golden Bridge, ZLI‐9506) and baked at 50°C for 30 min. Sections were washed twice with PBS, followed by antigen retrieval in citrate buffer (Beyotime, P0088) at 95°C for 30 min. After cooling to room temperature, sections were blocked in PBS containing 5% donkey serum (Yeasen, 36116ES10) and 0.3% Triton X‐100 for 1 h at room temperature, then incubated with primary antibodies (Table S1) overnight at 4°C. Following PBS washes, sections were incubated with appropriate secondary antibodies (Table S1) for 2 h at room temperature.

For cultured cells, coverslips were fixed with 4% PFA at 4°C for 30 min. After washing three times with PBS, cells were blocked and incubated with antibodies as described above.

Nuclei were counterstained with DAPI (Sigma–Aldrich, F6057). Images were acquired on a Leica Stellaris 5 confocal microscope (Leica Microsystems, Germany), with cortical regions captured using a 20× objective and detailed cellular morphology on coverslips imaged with a 40× oil‐immersion objective.

### 6.10 Chromatin Immunoprecipitation (ChIP‐qPCR) Assay

Chromatin immunoprecipitation (ChIP) assays were performed using the SimpleChIP Enzymatic Chromatin IP Kit (Magnetic Beads) (Cell Signaling Technology, 9003) according to the manufacturer's instructions. Briefly, cells were cross‐linked with 1% formaldehyde, and the reaction was quenched with glycine. Nuclei were isolated from lysed cells, and chromatin was digested with Micrococcal Nuclease (10011) at 37°C for 20 min to generate DNA fragments of 150–900 bp, followed by sonication to disrupt the nuclear membrane. Chromatin concentration and fragment size were validated, and 5–10 µg of chromatin DNA was incubated overnight at 4°C with specific antibodies or control IgG. ChIP‐Grade Protein G Magnetic Beads (9006) were used to capture immune complexes. After sequential washes with low‐ and high‐salt buffers, chromatin was eluted and reverse cross‐linked at 65°C. The recovered DNA was treated with Proteinase K, purified using spin columns, and analyzed by qPCR with gene‐specific primers to quantify the enrichment of target proteins at designated genomic loci.

### 6.11 Behavioral Assays

Behavioral tests were conducted using age‐matched male mice (12–14 weeks old). Mice were group‐housed (3–5 per cage) under standard conditions at 20–24°C with a 12‐h light/12‐h dark cycle (lights on from 09:00 to 21:00) and provided ad libitum access to food and water. All behavioral experiments were performed in a blinded manner, with investigators unaware of the animals’ genotypes.

### 6.12 Morris Water Maze (MWM) Test

The Morris water maze (MWM) test was performed in a circular black stainless‐steel pool (1.5 m in diameter). A circular escape platform (10 cm in diameter) was positioned in a fixed location. Mice were habituated to the testing environment for 1 h prior to behavioral assessment. The test consisted of three sequential phases: 1) a spatial acquisition (hidden platform) task, 2) a spatial probe trial to evaluate memory retention, and 3) a visible platform test to control for potential differences in visual acuity or motor function.

### 6.13 Hidden Platform Test

The water maze tracking software was initialized prior to testing, and the field of view was adjusted to encompass the entire pool surface. Animal identification numbers and group assignments were entered into the system. At the beginning of each trial, mice were placed into the water facing the pool wall from one of four starting positions, after which the experimenter immediately left the testing area. Swimming trajectories and latency to reach the platform were automatically recorded by the tracking system. The trial duration was set to 60 s. Upon locating the platform, mice were allowed to remain on it for 30 s. If a mouse failed to locate the platform within the allotted time, it was gently guided to the platform and allowed to remain there for the same duration to ensure equal exposure to spatial cues. Each mouse underwent three training trials per day with an inter‐trial interval of 20 min for five consecutive days. The platform position remained fixed throughout the training period. After each trial, mice were gently dried with a towel, briefly warmed, and returned to their home cages. Escape latency, swimming path length, and average swimming speed were quantified using the automated behavioral analysis system.

### 6.14 Probe Trial

Twenty‐four hours after the acquisition phase concluded, the hidden platform was removed to conduct the probe trial. Mice were released from a starting point in the quadrant opposite the original platform location and allowed to swim freely for 60 s. Swimming trajectories were recorded using an automated behavioral tracking system. Spatial memory was assessed by quantifying the time spent in the target quadrant and the number of platform crossings during the trial.

### 6.15 Visible Platform Test

The platform was elevated 1.5 cm above the water surface and marked with a distinct visual cue. Mice were released into the pool and allowed to swim until they located and mounted the platform. To prevent spatial bias, the test was repeated with the platform shifted to varying locations.

Behavioral data were recorded and analyzed using EthoVision XT software (Noldus Information Technology, Wageningen, the Netherlands).

### 6.16 Novel Object Recognition Test

The novel object recognition test relies on the innate tendency of rodents to preferentially explore novel objects over familiar ones. The apparatus consisted of an open‐field arena (50 × 50 × 30 cm). A video camera was mounted above the arena to track animal behavior, and all data were recorded and analyzed using EthoVision XT software. Prior to the sample phase, mice were habituated to the empty arena for 10 min per day for two consecutive days. During the sample (acquisition) phase, two identical objects (A1 and A2) were placed in adjacent corners of the arena, approximately 15 cm from the walls. Mice were placed in the center of the arena facing away from the objects and allowed to explore freely for 4 min. Following this session, animals were returned to their home cages for a 2 h retention interval. During the test phase, one copy of the familiar object (A3) and one novel object (B) were placed in the locations previously occupied by A1 and A2. Mice were placed in the center of the arena facing away from the objects and allowed to explore freely for 3 min. The time spent exploring each object was recorded. After each trial, mice were returned to their home cages, and the arena was thoroughly cleaned with 75% ethanol to eliminate residual odors before testing the next animal.

Data Analysis: Exploration time was defined as the duration during which the mouse directed its nose toward an object at a distance of < 2 cm. The Recognition Index (RI) was calculated as:

$$RI=\frac{Tnovel}{Tnovel+Tfamiliar}\times 100\%$$

The Discrimination Index (DI) was calculated using the formula:

$$DI=\left(\frac{Tnovel}{Ttotal}\times 100\%\right)-\left(\frac{Tfamiliar}{Ttotal}\times 100\%\right)$$

Higher RI and DI values indicate better memory retention for the novel object. All experimental procedures were video‐recorded and scored by an investigator blinded to genotype and treatment to ensure objective assessment.

### 6.17 Y‐Maze Spontaneous Alternation Test

The Y‐maze apparatus consisted of three identical arms (30 × 10 × 10 cm) positioned at 120° angles relative to each other. A video camera was mounted above the maze for automated behavioral tracking. Prior to testing, detection parameters were calibrated using a test animal to ensure accurate tracking. Mice were transported to the testing room and allowed to habituate to the environment for 60 min before the experiment. At the start of each trial, a mouse was placed at the central junction of the maze facing one arm and allowed to explore freely for 10 min. To minimize external disturbances, the experimenter remained quiet and at a distance from the apparatus throughout the test. After each trial, mice were returned to their home cages, and the maze was thoroughly cleaned with 75% ethanol to eliminate residual odors before testing the next animal.

### 6.18 Data Analysis

The number and sequence of arm entries were recorded and analyzed using EthoVision XT software. Spontaneous alternation behavior was defined as consecutive entries into all three different arms. The percentage of spontaneous alternation was calculated using the following formula:

$$Alternation\left(\%\right)=\frac{Numberofactualalternations}{Total\;arm\;entries-\;2}\times 100$$

Higher spontaneous alternation rates indicate better spatial working memory.

### 6.19 Open Field Test

The Open Field Test was conducted in a square arena measuring 50 × 50 × 30 cm. The floor was digitally divided into three zones: a central zone (20 × 20 cm), a transition zone, and a peripheral zone. A video camera was mounted above the arena to record behavioral data. Detection parameters were calibrated prior to testing to ensure accurate tracking. Mice were transported to the testing room and allowed to habituate to the environment for 60 min before testing. The arena was cleaned with 75% ethanol between trials. Each mouse was placed in the center of the arena, and its behavior was recorded for 10 min. To minimize stress, the experimenter remained quiet and at a distance from the apparatus during the test. After testing, mice were returned to their home cages, and the arena was thoroughly cleaned with 75% ethanol to remove residual odors. Behavioral parameters—including total distance traveled, average velocity, time spent in the center zone, and the number of entries into the center zone—were quantified using EthoVision XT software.

### 6.20 RNA Sequencing and Data Analysis

Total RNA was extracted from three independent biological replicates of NSD2‐knockout and control NSCs. High‐throughput RNA sequencing was performed by Beijing CapitalBio Technology Co., Ltd. (Beijing, China). Paired‐end reads were generated on the Illumina platform, and the quality of raw reads was initially assessed using FastQC (v0.11.2) [45]. Low‐quality sequences and adapter contamination were subsequently removed using fastp (v0.14.0) [46]. Clean reads were aligned to the reference genome using HISAT2 (v2.2.0) [47], and mapping quality was evaluated with RSeQC (v2.6.4) [48]. Transcript assembly and quantification were conducted with StringTie (v2.2.1) [49], while featureCounts (subread‐2.0.0) [50] was used to generate read counts per gene for downstream differential expression analysis. Differentially expressed genes (DEGs) were identified using DESeq2 (v1.46.0) [51], with thresholds set at FDR < 0.05 and |log_2_(fold change)| > 0.585. Functional enrichment analyses, including Gene Ontology (GO) and Kyoto Encyclopedia of Genes and Genomes (KEGG) pathway analyses, were performed using clusterProfiler (v4.14.6) [52]. Additionally, Gene Set Enrichment Analysis (GSEA, v4.4.0) [53] was conducted to assess coordinated expression changes of predefined gene sets across the transcriptome.

### 6.21 CUT&Tag Library Construction and Data Analysis

Protein‐DNA interactions were profiled using the Hyperactive Universal CUT&Tag Assay Kit for Illumina Pro (TD904; Vazyme, Nanjing, China), following the manufacturer's standard protocol. Briefly, approximately 1.4 × 10^5^ cells were harvested per reaction and bound to Concanavalin A (ConA)‐coated magnetic beads. Cell membranes were permeabilized with digitonin, followed by sequential incubation with a target‐specific primary antibody and a secondary antibody. Targeted DNA cleavage and adapter tagging were mediated by a high‐activity Protein A/G‐fused transposase (pA/G‐Tnp Pro). The tagmentation reaction was performed at 37°C for 60 min, generating DNA fragments flanked by sequencing adapters. Tagged DNA fragments were subsequently extracted using magnetic beads and subjected to PCR amplification. The number of PCR cycles was optimized according to the initial cell input and target protein abundance. PCR products were purified using VAHTS DNA Clean Beads, yielding final sequencing libraries compatible with the Illumina platform. Raw sequencing reads were first processed to remove low‐quality sequences and adapter contamination, generating high‐quality Clean Reads. These reads were aligned to the reference genome using Bowtie2 [54], and only uniquely mapped reads were retained for downstream analyses. Multi‐mapping reads and PCR duplicates were excluded to ensure accuracy and reliability. Enriched genomic regions were identified from the unique reads using MACS2 [55]. The distribution of peaks across genomic features (promoters, gene bodies, intergenic regions) and chromosomes was assessed. Genome browser tracks and average signal profiles were generated with deepTools to visualize protein enrichment at specific genomic loci [56]. Differential binding analysis was conducted with the DiffBind package to assess occupancy changes across experimental conditions. To infer the biological roles of the target protein, Gene Ontology (GO) and KEGG pathway enrichment analyses were performed on the peak‐associated genes. Motif analysis was conducted using MEME to identify overrepresented DNA‐binding motifs within the peak regions [57].

### 6.22 Statistical Analysis

All data are presented as mean ± SEM. Statistical analyses were performed using GraphPad Prism 10.1.2. For all two‐group comparisons, data distribution was first assessed using the Shapiro‐Wilk test. Homogeneity of variance was evaluated using F‐test. For datasets that followed a normal distribution, unpaired two‐tailed Student's *t*‐tests were used when variances were equal, and Welch's *t*‐tests were applied when variances were unequal. Statistical significance was defined as *p* < 0.05. Detailed statistical information, including normality testing, variance analysis, the specific statistical test used for each comparison, and exact *p* values, is provided in Table S2.

## Author Contributions

All authors participated in the scientific discussion. **X.P**., **P.S**., and **R.G**. conceived and directed the project, including the experimental design and data interpretation. **H.C**. and **M.L**. carried out the experimental studies and analyses. **L.H**. and **B.Y**. contributed to some experiments for cloning and data analysis. **H.C**. and **P.S**. wrote the manuscript. **X.P**., **P.S**., **R.G**., and **B.Q**. revised the manuscript. All authors commented on the manuscript. All the authors have read and approved the final manuscript.

## Funding

This work was supported by National Science and Technology Innovation 2030 Grants of China (2021ZD0200900 to X.P.), National Key Research and Development Program of China (2022YFA1103803 to X.P.), Prevention and Control of Emerging and Major Infectious Diseases‐National Science and Technology Major Project (2025ZD01900700 to R.G.), National Natural Science Foundation of China (32370883 to P.S.), CAMS Innovation Fund for Medical Sciences (CIFMS; 2021‐I2M‐1‐019 to P.S. and 2021‐I2M‐1‐024 to X.P.), Beijing Natural Science Foundation (5262021 to P.S.), State Key Laboratory Special Fund 2060204.

## Ethics Statement

The animal studies were performed in accordance with the guidelines and were approved by the Institutional Animal Care and Use Committee (IACUC) of the Chinese Academy of Medical Sciences & Peking Union Medical College (Approval No. GR24002).

## Conflicts of Interest

The authors declare no conflicts of interest.

## Supporting information




**Supporting File 1**: advs76695‐sup‐0001‐SuppMat.docx.

**Supporting File 2**: advs76695‐sup‐0002‐TableS1.xlsx.

**Supporting File 3**: advs76695‐sup‐0003‐TableS2.xlsx.

## Data Availability

The raw sequence data reported in this paper have been deposited in the Genome Sequence Archive (Genomics, Proteomics & Bioinformatics 2025) in National Genomics Data Center (Nucleic Acids Res 2025), China National Center for Bioinformation / Beijing Institute of Genomics, Chinese Academy of Sciences (GSA: CRA044655) that are publicly accessible at https://ngdc.cncb.ac.cn/gsa [58, 59]. Additionally, the ChIP‐seq data presented in Figure 5H of this paper were cited in the published study: *Loss of NSD2 causes dysregulation of synaptic genes and altered H3K36 dimethylation in mice*. The dataset is available under accession number GSE232566. Supporting Information is available online.
