# Supplementary material for: NSD2 Coordinates the Neurogenic‐to‐Gliogenic Transition via H3K36me2‐Dependent Activation of the EGFR‐ERK Pathway
Source: Adv Sci (Weinh). 2026 Jul 27:e76695. Online ahead of print. doi: 10.1002/advs.76695 (PMC13403718; doi:10.1002/advs.76695)
Supplement: Supplementary file 1 — Supporting File 1: advs76695‐sup‐0001‐SuppMat.docx. [file ADVS-9999-e76695-s003.docx]

**NSD2 Coordinates the Neurogenic-to-Gliogenic Transition via H3K36me2-Dependent Activation of the EGFR-ERK Pathway**

Hanxue Chen^1#^, Mengyuan Li^3#^, Lin Hou^1^, Bin Yin^1^, Boqin Qiang^1^, Ran Gao^3*^, Pengcheng Shu^1*^ and Xiaozhong Peng^1,2*^

**This file includes:**

Supplementary Figure S1-S13

**
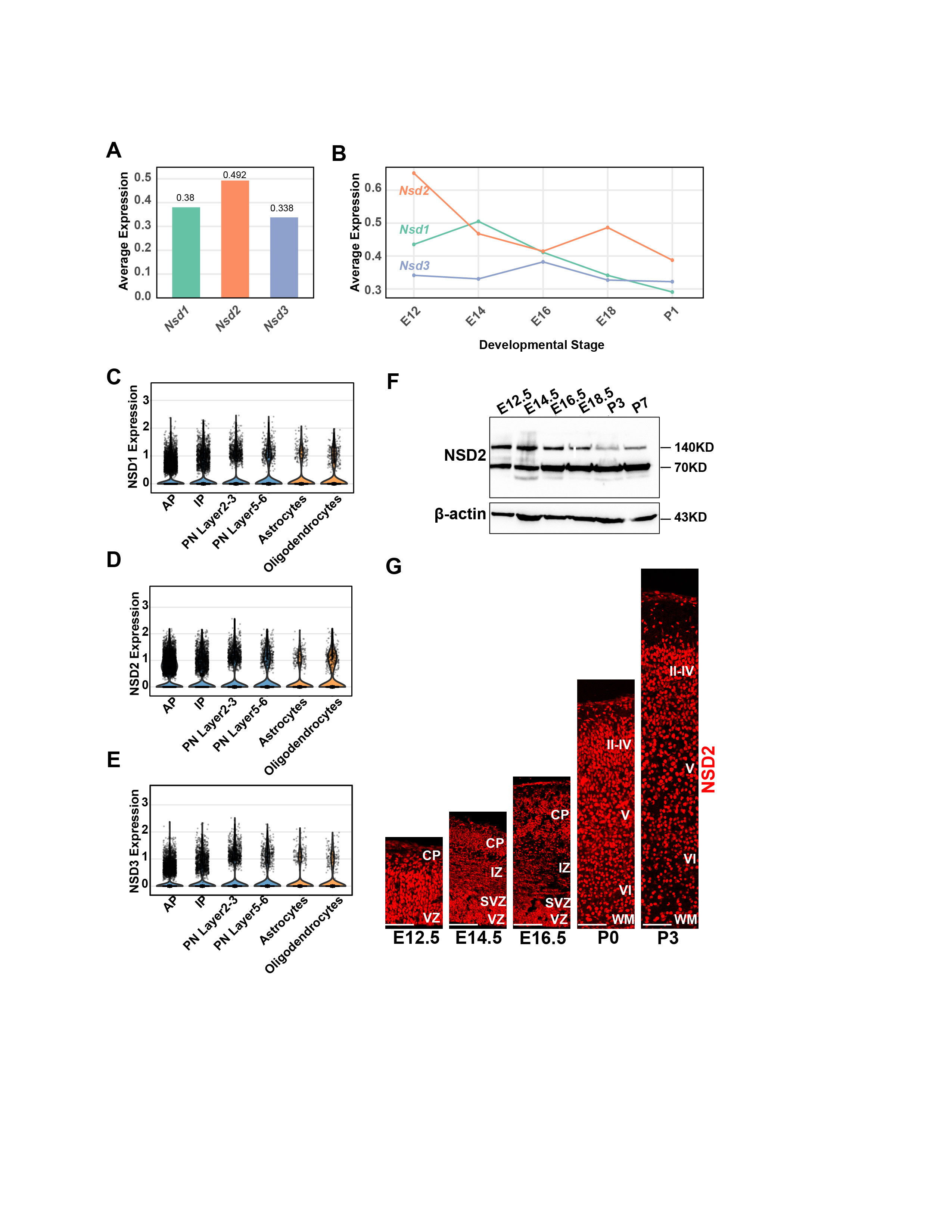
**

**Figure S1. NSD2 is broadly expressed in the developing mouse neocortex.** (A and B) Analysis of *Nsd* family member expression in the developing mouse cortex using a publicly available scRNA-seq dataset (Di Bella et al., *Nature* 2021). Shown are the overall mean expression levels (A) and the temporal expression trajectories from E12.5 to P1 (B). (C–E) Comparative analysis of *Nsd* family expression across distinct cell populations, including intermediate progenitors (IP), layer 2/3 and layer 5/6 cortical projection neurons (PN L2/3, PN L5/6), apical progenitors (AP), astrocytes, and oligodendrocytes. (F) Western blot analysis showing NSD2 protein abundance in the isolated dorsal cortex of mice from embryonic day 12.5 (E12.5) to postnatal day 7 (P7). Note that both the ~140 kDa and ~70 kDa bands represent the NSD2 protein. The same results were observed in the following experiments. (G) Representative immunofluorescence images illustrating the spatiotemporal expression pattern of NSD2 protein in the neocortex at various developmental stages (E12.5 to P3). Abbreviations: CP, cortical plate; IZ, intermediate zone; SVZ, subventricular zone; VZ, ventricular zone; WM, white matter.

**
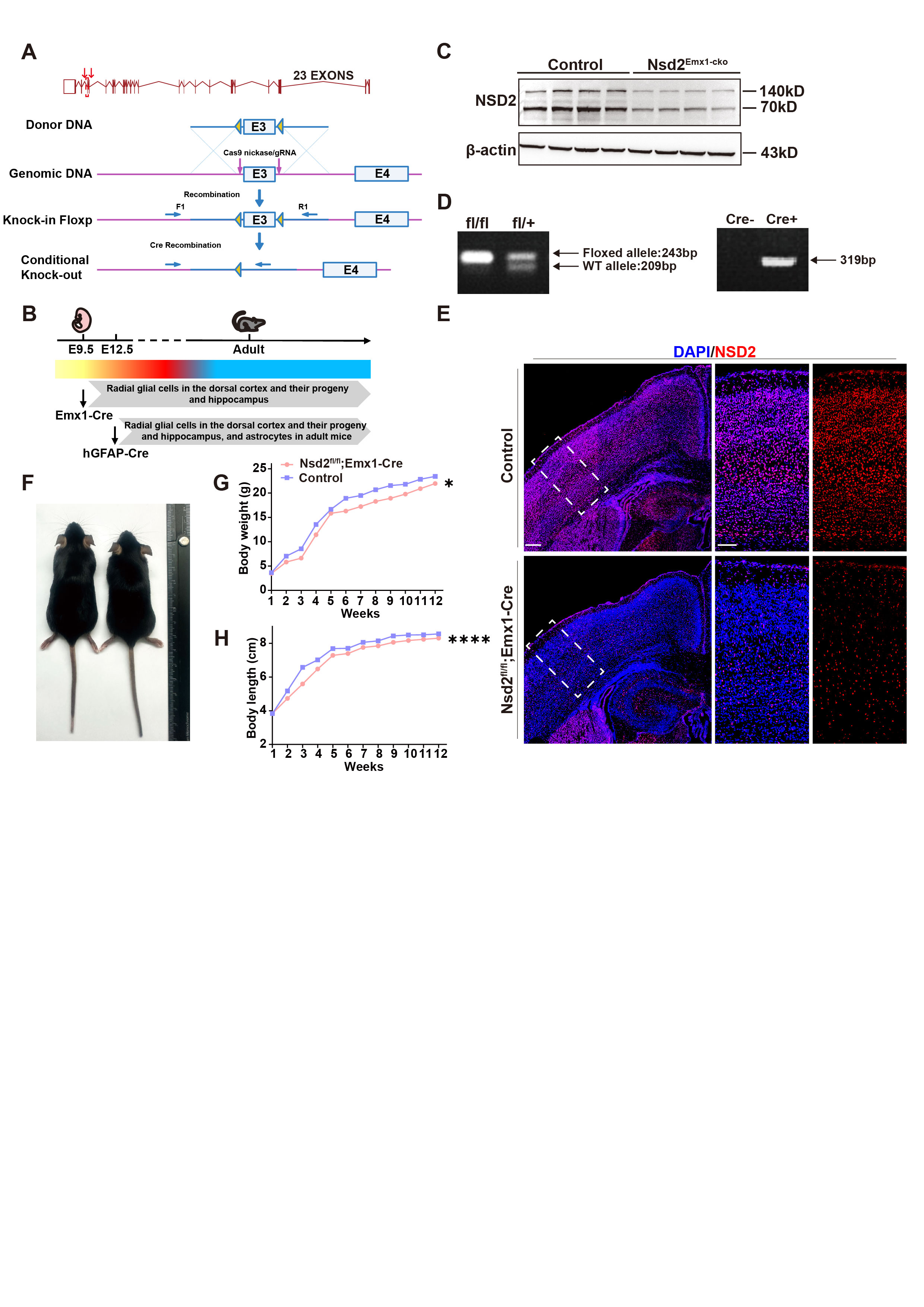
**

**Figure S2. Generation and validation of *Nsd2* conditional knockout mice.** (A) Schematic diagram illustrating the strategy for generating *Nsd2* conditional knockout (cKO) mice. *LoxP* sites were inserted flanking exon 3 of the *Nsd2* gene to mediate Cre-dependent excision. (B) Schematics showing the expression domains of *Emx1-Cre* and *hGFAP-Cre* drivers used in this study. (C) Western blot analysis verifying NSD2 protein depletion in the P3 dorsal cortex. Note: The residual NSD2 signal in the cKO lysate is likely attributable to minor contamination from ventral cortical tissues where *Emx1-Cre* is not active. (D) Genotyping of embryonic mice via PCR analysis of tail genomic DNA. (E) Representative immunofluorescence images of P3 coronal sections confirming the loss of NSD2 protein in the dorsal cortex. Scale bar, 200 µm. (F–H) Monitoring of body weight and body length trajectories in control and *Nsd2*-cKO mice from 1 to 12 weeks of age.

For (F–H), n = 10 animals per group. Statistical significance was determined using two-way ANOVA. *P < 0.05, **P < 0.01, ***P < 0.001, ****P < 0.0001. Data are presented as mean ± SEM.


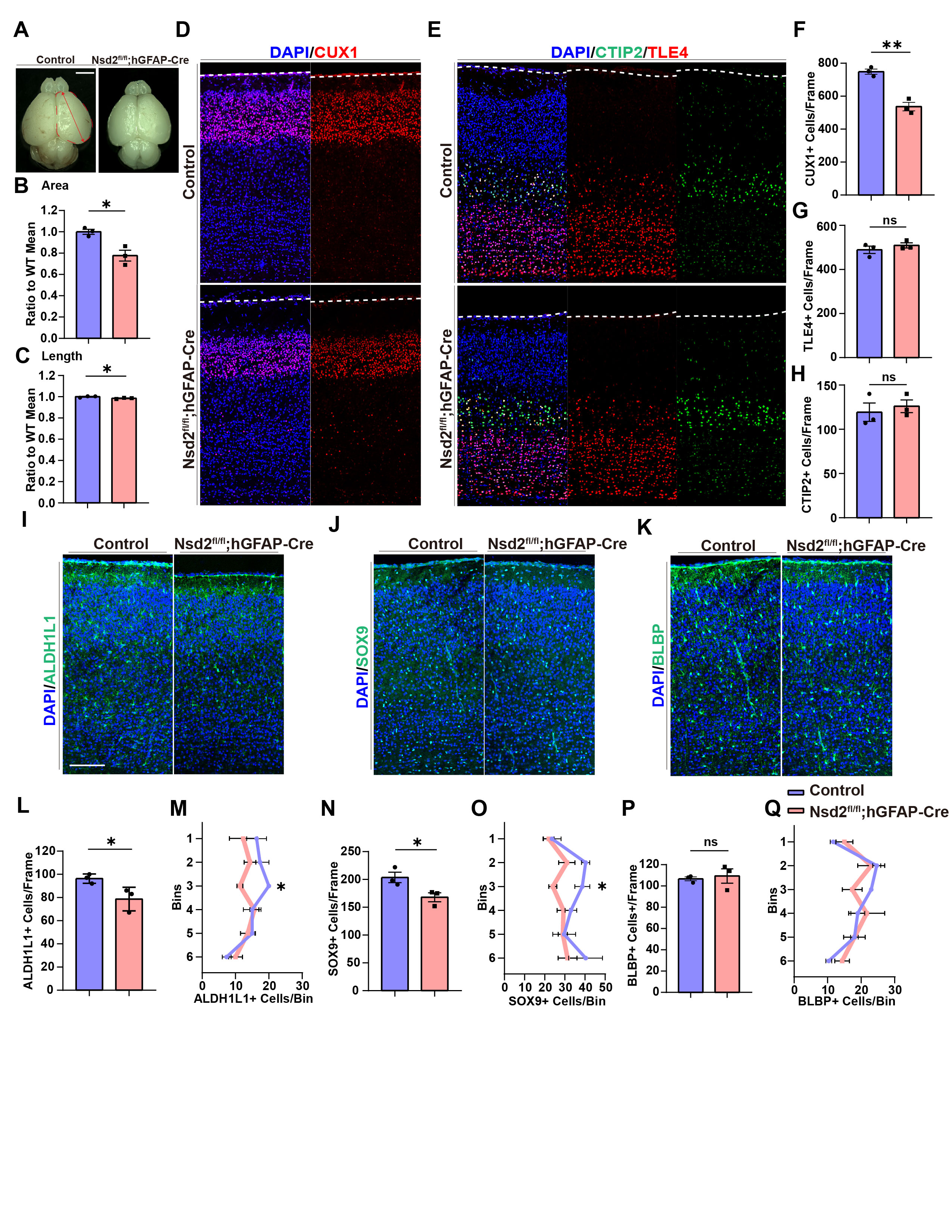


**Figure S3. *Nsd2^hGFAP^*^-cKO^ mice recapitulate neuronal and astrocytic defects.** (A) Stereomicroscopic images of P3 *Nsd2^hGFAP^*^-cKO^ mice brains following PFA perfusion. Red dashed lines and arrows indicate the measured area and length of the telencephalon, respectively. Scale bar, 2 mm. (B, C) Quantitative analysis of the cortical area(B) and length(C) shown in (A) (n = 3). (D, E) Representative immunofluorescence images of the upper-layer projection neuron marker CUX1(D) and the deep-layer marker TLE4 and CTIP2(E) in the P3 neocortex. (F-H) Quantification of total CUX1⁺ (F) and TLE4⁺ (G) and CTIP2^+^ (H) neuron densities. (I–K) Representative immunofluorescence images of astrocyte markers ALDH1L1 (I), SOX9 (J), and BLBP (K) in the P3 neocortex. (L–Q) Cortical sections were divided into six equal bins from the ventricular surface (Bin 6) to the pial surface (Bin 1). Quantification of total cell densities and laminar distributions (six bins) for ALDH1L1⁺ (L, M), SOX9⁺ (N, O), and BLBP⁺ (P, Q) astrocytes.

Scale bar, 200 µm. Data are presented as mean ± SEM. Statistical significance was determined using an unpaired Student’s t-test or Welch’s t-test for total counts (B, C, F-H, L, N, P) and two-way ANOVA with Sidak’s multiple comparisons test for bin analyses (M, O, Q). *P < 0.05, **P < 0.01, ***P < 0.001, ****P < 0.0001.


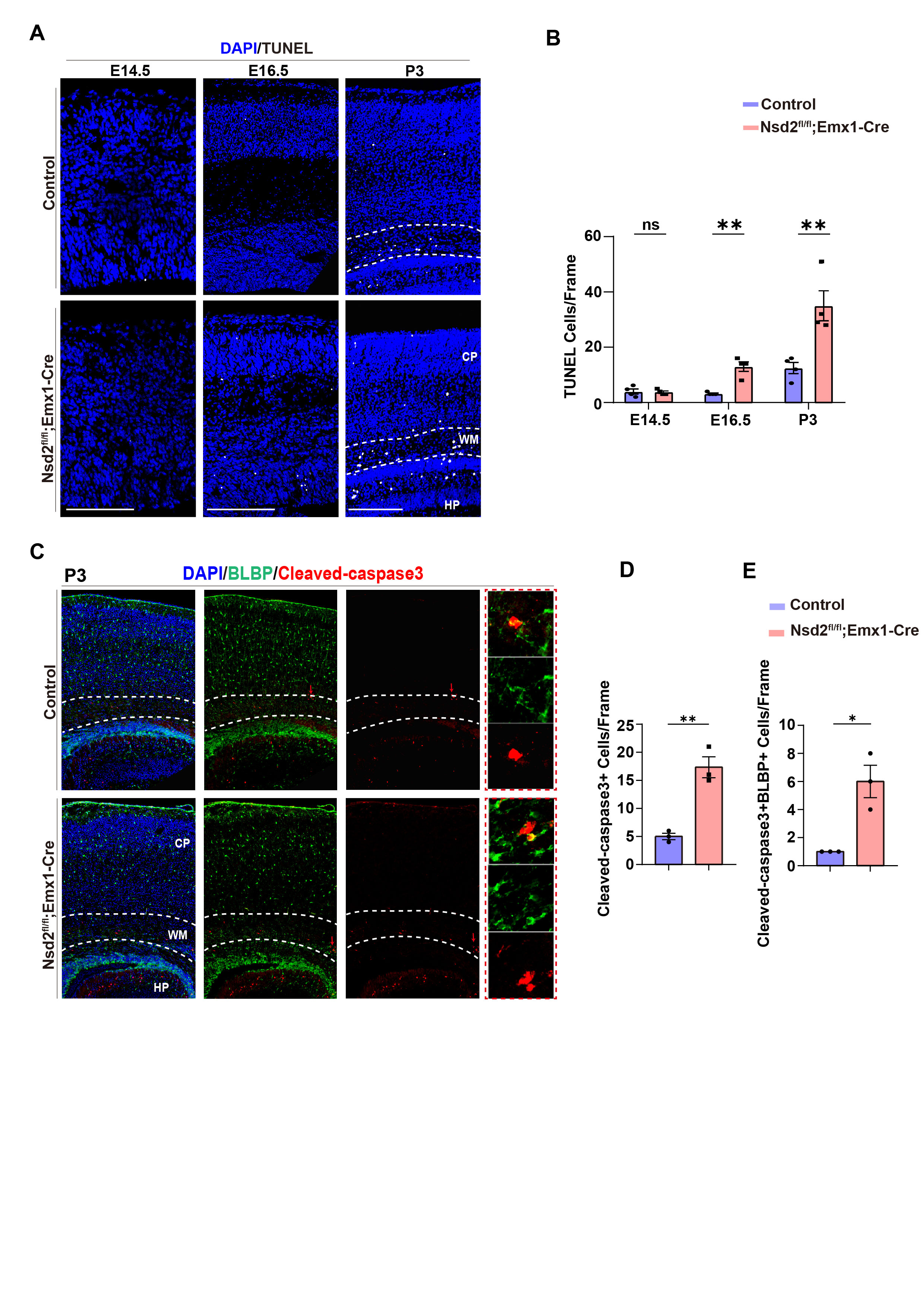


**Figure S4. Increased Apoptosis in *Nsd2^Emx1^*^-cKO^ Mice.** (A) Representative images of TUNEL staining in the neocortex at E14.5, E16.5, and P3, labeling apoptotic cells. (B) Quantification of the density of TUNEL^+^ cells at the indicated time points. (C) Representative immunofluorescence images of cleaved-caspase-3 (CASP3) and BLBP co-staining in the neocortex of control and Nsd2^Emx1-cKO^ mice at P3. Red boxes and arrows indicate representative CASP3⁺BLBP⁺ double-positive cells. (D) Quantification of CASP3^+^ cells in control and Nsd2^Emx1-cKO^ cortices. **(E)** Quantification of CASP3^+^BLBP^+^ double-positive cells in control and Nsd2^Emx1-cKO^ cortices.

Scale bar, 200 µm. Data are presented as mean ± SEM. Statistical significance was determined using an unpaired Student’s t-test or Welch’s t-test for total counts. *P < 0.05, **P < 0.01, ***P < 0.001, ****P < 0.0001. Abbreviations: WM, white matter; HP, Hippocampus.


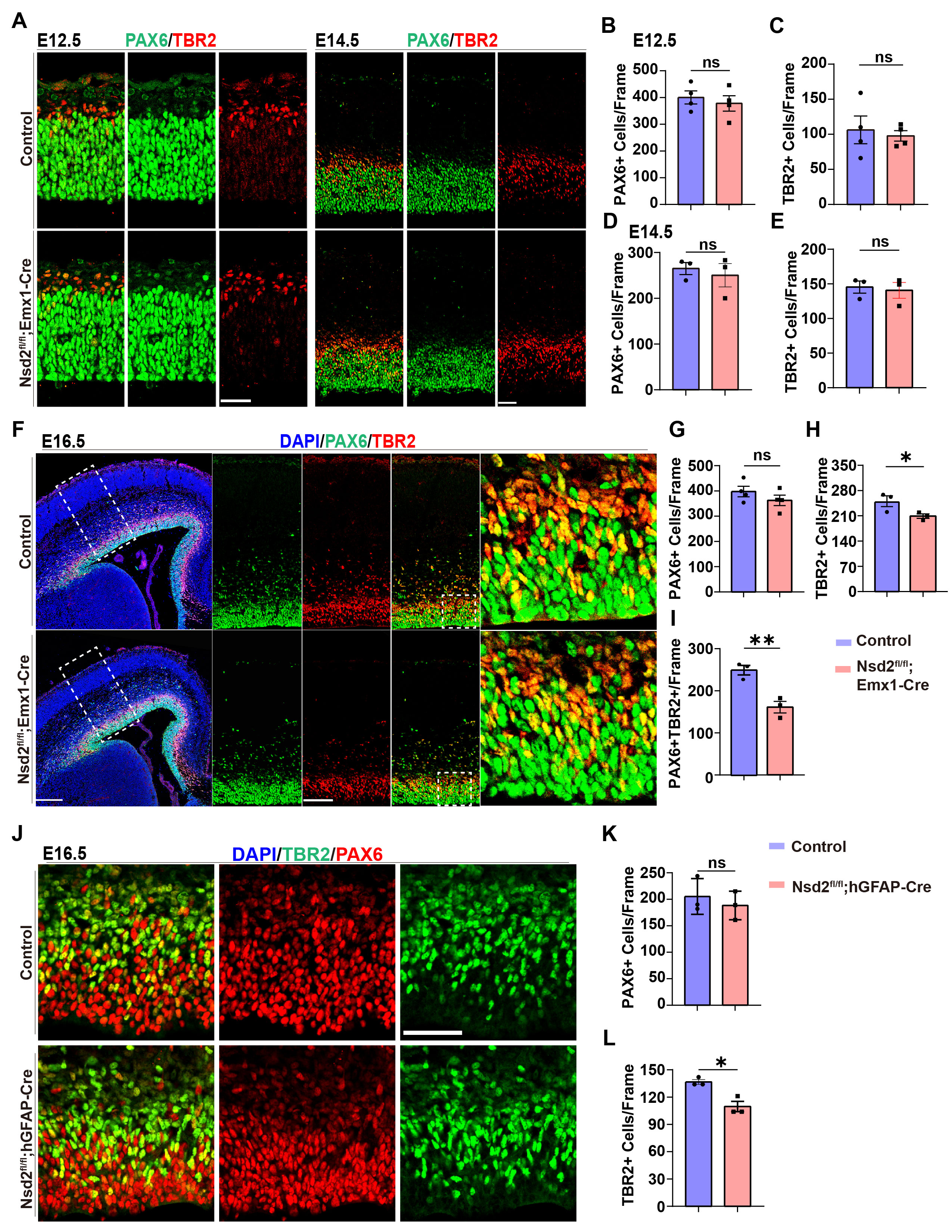


**Figure S5. Altered Fate of Late Embryonic Progenitors in *Nsd2* Knockout Mice.** (A) Representative immunofluorescence staining of the radial glial cell (RGC) marker PAX6 and the intermediate progenitor (IP) marker TBR2 in the *Nsd2*^Emx1-cKO^ neocortex at E12.5 and E14.5. (B, C) Quantification of PAX6^+^ (B) and TBR2^+^ (C) cells at E12.5. (D, E) Quantification of PAX6^+^ (D) and TBR2^+^ (E) cells at E14.5. (F) Representative immunofluorescence images of PAX6 and TBR2 staining in the neocortex of control and *Nsd2*^Emx1-cKO^ mice at E16.5. (G-I) Quantification of PAX6^+^ (G) and TBR2^+^ (H), and PAX6^+^TBR2^+^ (I) cells at E16.5. (J) Representative immunofluorescence images of PAX6 and TBR2 staining in the neocortex of control and *Nsd2^hGFAP-cKO^* mice at E16.5. (K, L) Quantification of PAX6^+^ (K) and TBR2^+^ (L) cells at E16.5.

Scale bar, 100 µm. Data are presented as mean ± SEM. Statistical significance was determined using an unpaired Student’s t-test or Welch’s t-test for total counts. *P < 0.05, **P < 0.01, ***P < 0.001, ****P < 0.0001.


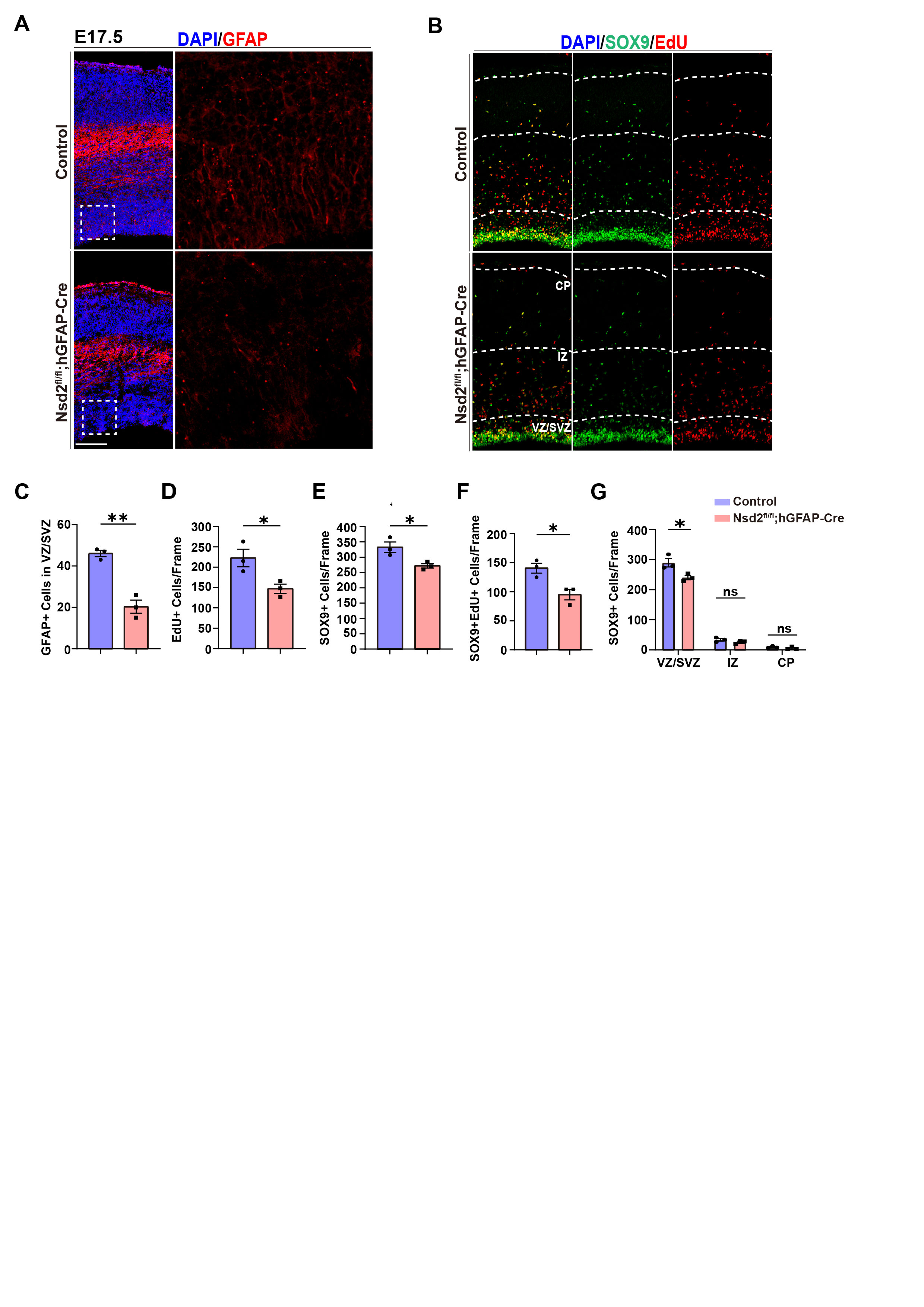


**Figure S6. *Nsd2*^hGFAP-cKO^ mice recapitulate gliogenic defects. (A)** Representative immunofluorescence images of GFAP staining in the neocortex of control and *Nsd2*^hGFAP-cKO^ mice at E17.5. **(B)** Representative images of SOX9 and EdU co-staining following a 2 h EdU pulse-labeling assay at E17.5. **(C)** Quantification of GFAP^+^ cells in the VZ/SVZ. **(D)** Quantification of EdU^+^ cells. **(E)** Quantification of SOX9^+^ cells. **(F)** Quantification of SOX9^+^EdU^+^ double-positive cells. **(G)** Quantification of SOX9^+^ cells in the VZ/SVZ, IZ, and CP.

Scale bars, 100 μm. Data are presented as mean ± SEM. n = 3 mice per group. Unpaired Student’s t-test or Welch’s t-test was used as appropriate. *P < 0.05, **P < 0.01, ***P < 0.001, ****P < 0.0001.

**
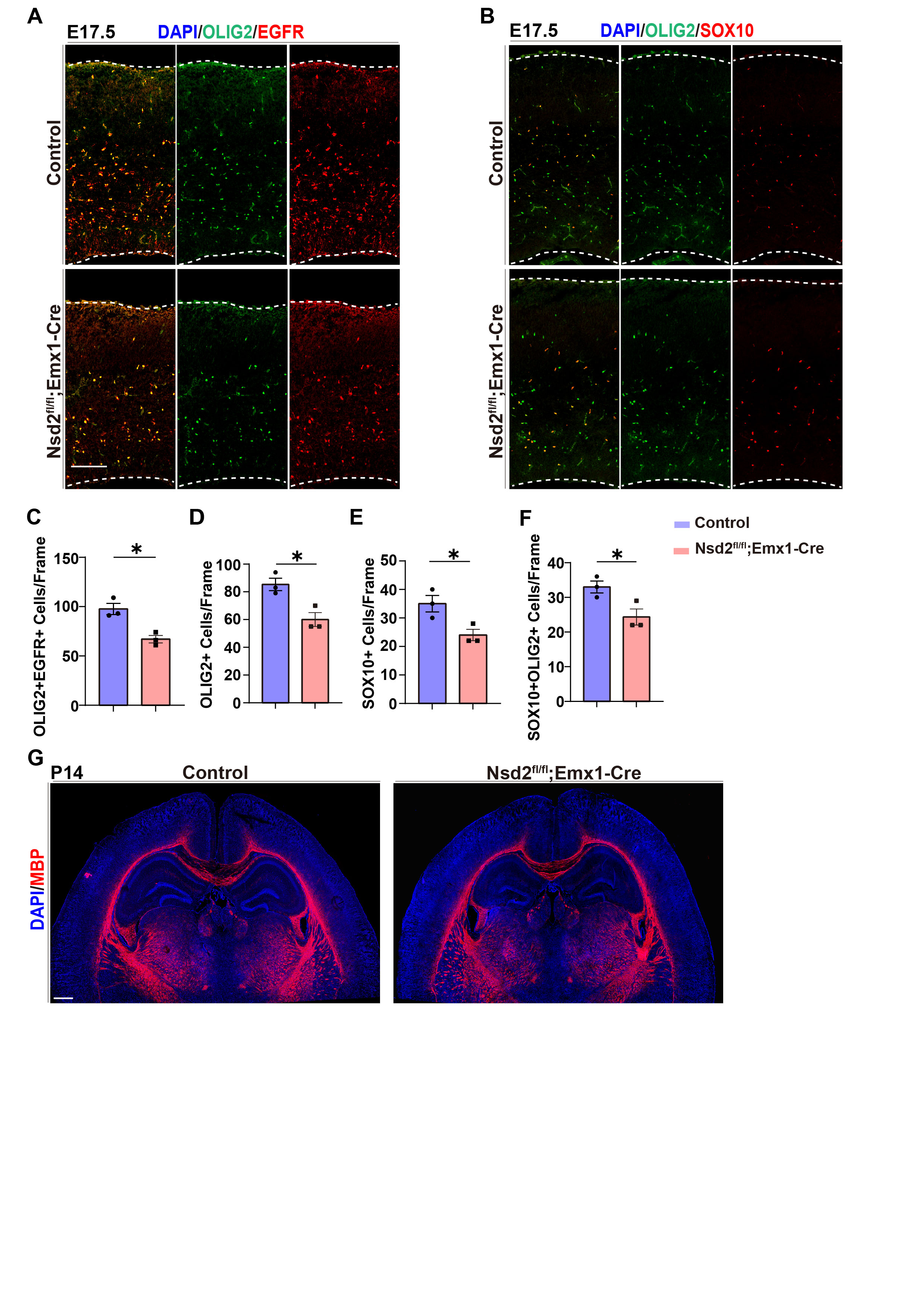
**

**Figure S7. Impaired oligodendrocyte lineage development in *Nsd2*^Emx1-cKO^ mice.**  (A) Representative immunofluorescence images of OLIG2 (green) and EGFR (red) co-staining in the neocortex at E17.5. OLIG2^+^EGFR^+^ double-positive cells represent EGFR-responsive OPCs. Scale bars, 100 μm. (B) Representative immunofluorescence images of OLIG2 (green) and SOX10 (red) co-staining in the neocortex of control and *Nsd2*^Emx1-cKO^ mice at E17.5. OLIG2 and SOX10 label oligodendrocyte lineage cells. (C) Quantification of OLIG2^+^EGFR^+^ double-positive cells. (D) Quantification of OLIG2^+^ cells in B. (E) Quantification of SOX10^+^ cells. (F) Quantification of OLIG2^+^SOX10^+^ double-positive cells. (G) Representative immunofluorescence images of MBP (Myelin Basic Protein) in the neocortex at P14. Scale bars, 500 μm.

Data are presented as mean ± SEM. n = 3 mice per group. Unpaired Student’s t-test or Welch’s t-test was used as appropriate. *P < 0.05, **P < 0.01, ***P < 0.001, ****P < 0.0001.


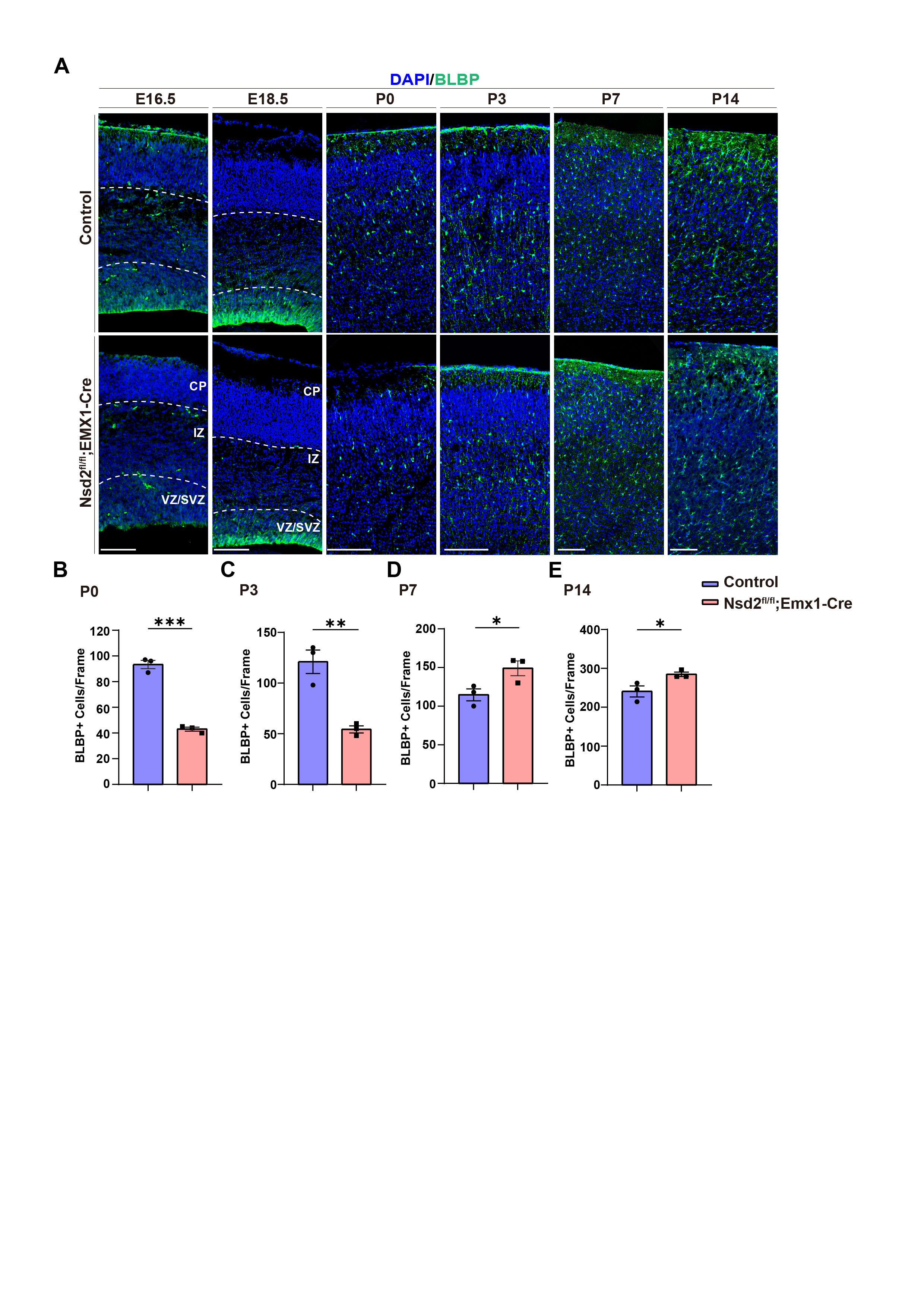


**Figure S8. Developmental time-course analysis of BLBP expression following *Nsd2* deletion.**  (A) Representative immunofluorescence images of BLBP in the neocortex of control and *Nsd2*^Emx1-cKO^ mice at the indicated developmental stages (E16.5, E18.5, P0, P3, P7, and P14). BLBP predominantly labels radial glial fibers at embryonic stages (E16.5 and E18.5) and immature astrocyte-lineage cells at postnatal stages (P0–P14). At E18.5, BLBP^+^ fibers in control cortices extended into the intermediate zone (IZ), whereas fiber extension was markedly reduced in *Nsd2*^Emx1-cKO^ cortices. (B-E) Quantification of BLBP^+^ cell density in the cortex of control and *Nsd2*^Emx1-cKO^ mice at P0 (B), P3 (C), P7 (D), and P14 (E). Note that the P0 and P3 datasets were also presented in Fig. 1F,G and Fig. 1I,J, respectively.

Scale bars: 100 µm (E16.5 and E18.5); 200 µm (P0-P14). Data are presented as mean ± SEM. n = 3 mice per group at each time point. Unpaired Student’s t-test or Welch’s t-test was used as appropriate. Detailed statistical information is provided in Supplementary Table S2. Quantification at P7 and P14 was performed within a cortical region measuring 800 µm × 1600 µm. *P < 0.05, **P < 0.01, ***P < 0.001, ****P < 0.0001.


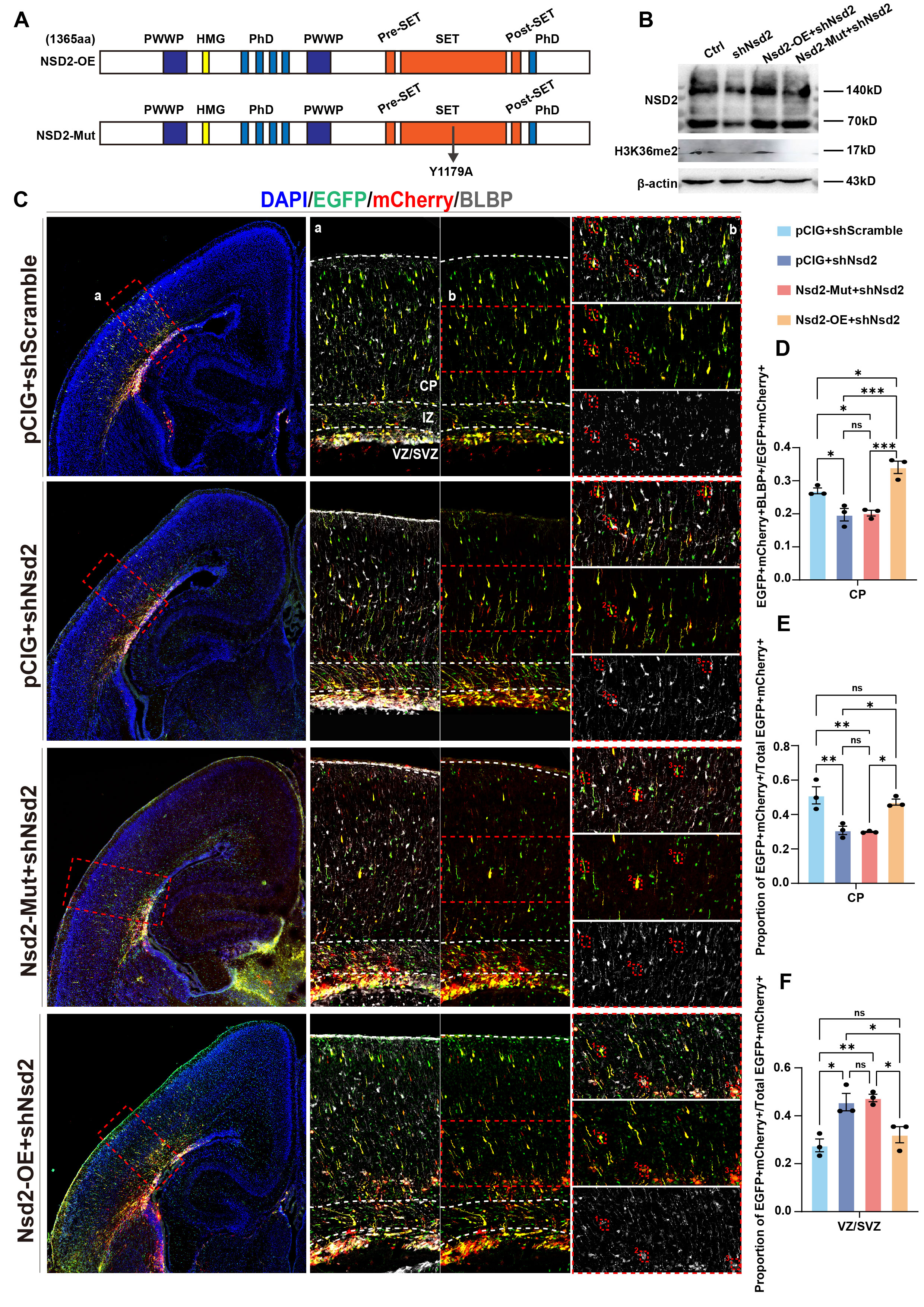


**Figure S9. NSD2 promotes astrocyte differentiation through its methyltransferase activity.** (A) Schematic representation of the NSD2-Y1179A mutant construct. The NSD2-Y1179A point mutation was generated at the tyrosine residue at position 1179 (Y1179) within the catalytic SET domain. This mutation substitutes the tyrosine with alanine, which is well-established to abolish its histone methyltransferase activity. (B) Validation of shNsd2, Nsd2-OE, and Nsd2-Mut expression by Western blot analysis. (C) Representative images of co-electroporated cells in the cortex following in utero electroporation at E15.5 and analysis at P0. Four experimental groups were included: shScramble + pCIG, shNsd2 + pCIG, shNsd2 + Nsd2-Mut, and shNsd2 + Nsd2-OE. Sections were co-stained for EGFP (green), mCherry (red), and BLBP (magenta). Insets indicate representative EGFP^+^mCherry^+^BLBP^+^ triple-positive cells. Note: The shRNA plasmids (shScramble or shNsd2) express mCherry, whereas the overexpression plasmids (empty vector, Nsd2-OE, or catalytically inactive Nsd2-Mut Y1179A) express EGFP. (D) Percentage of EGFP^+^mCherry^+^BLBP^+^ triple-positive cells among total EGFP^+^mCherry^+^ co-transfected cells in the CP. (E) Percentage of EGFP^+^mCherry^+^ cells located in the CP relative to total EGFP^+^mCherry^+^ co-transfected cells. (F) Percentage of EGFP^+^mCherry^+^ cells retained in the VZ/SVZ relative to total EGFP^+^mCherry^+^ co-transfected cells.

Data are presented as mean ± SEM. n = 3 embryos per group. One-way ANOVA followed by Tukey’s multiple-comparisons test was used for statistical analysis. *P < 0.05, **P < 0.01, ***P < 0.001, ****P < 0.0001.


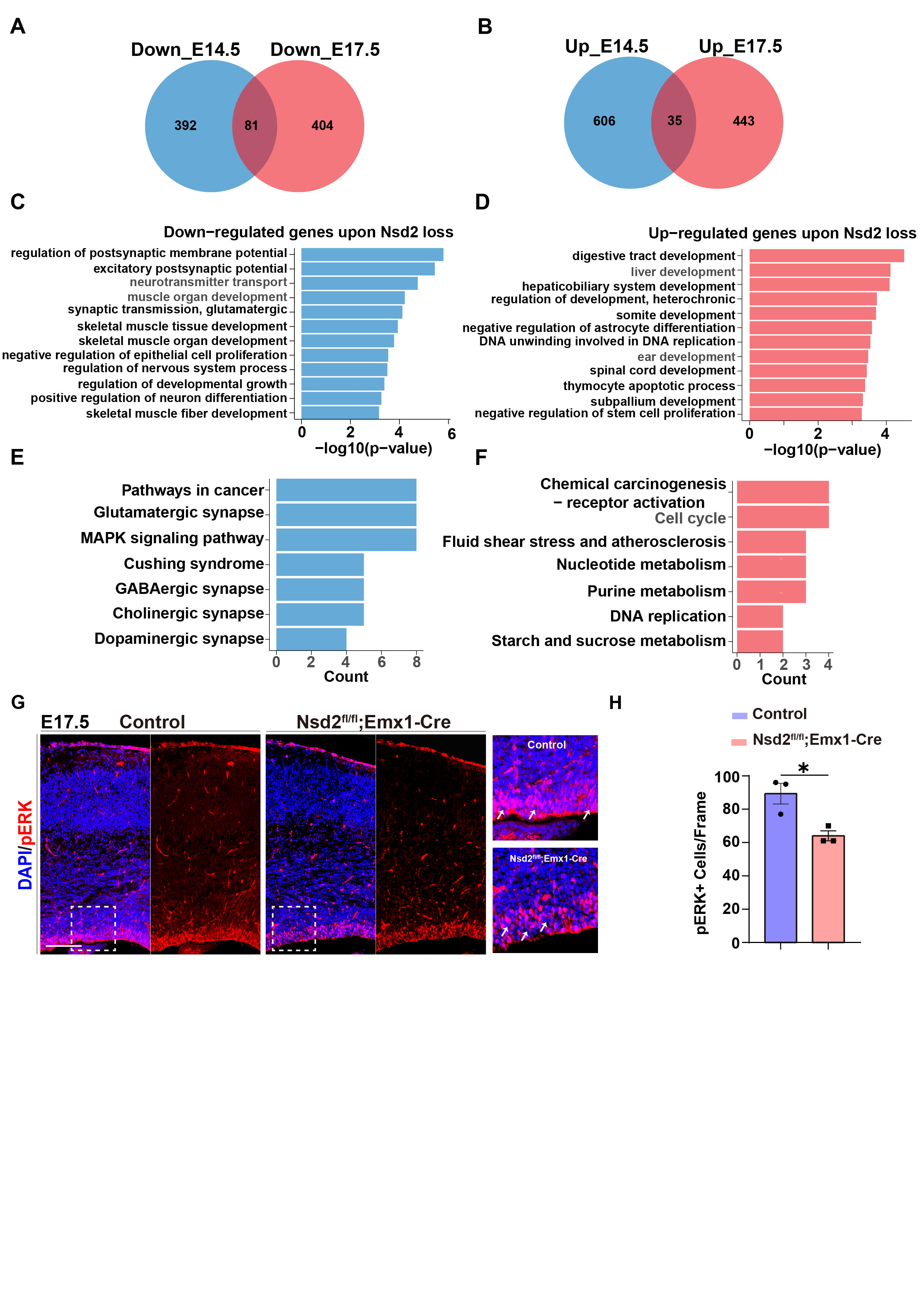


**Figure S10. Transcriptomic analysis of *Nsd2^Emx1^*^-cKO^ mice.** (A, B) Venn diagrams showing the overlap of downregulated genes (A) and upregulated genes (B) between E14.5 *Nsd2^Emx1^*^-cKO^ dorsal cortical tissues and E17.5 *Nsd2*-deficient NSCs. (C, D) Gene Ontology (GO) enrichment analysis of overlapping downregulated (C) and upregulated (D) genes identified in (A) and (B). (E, F) KEGG pathway enrichment analysis of overlapping downregulated (E) and upregulated (F) genes identified in (A) and (B). Data information: RNA-seq datasets were derived from three independent biological replicates per group for both WT and cKO mice. (G) Representative immunofluorescence images showing p-ERK expression in cortices of E17.5 *Nsd2^Emx1^*^-cKO^ mice. Scale bar, 100 μm (H) Quantification of p-ERK^+^ cell density from the experiment in (G). Data are presented as mean ± SEM. n = 3 embryos per group. Statistical significance was determined by Unpaired t test with Welch's correction. *P < 0.05, **P < 0.01, ***P < 0.001, ****P < 0.0001.


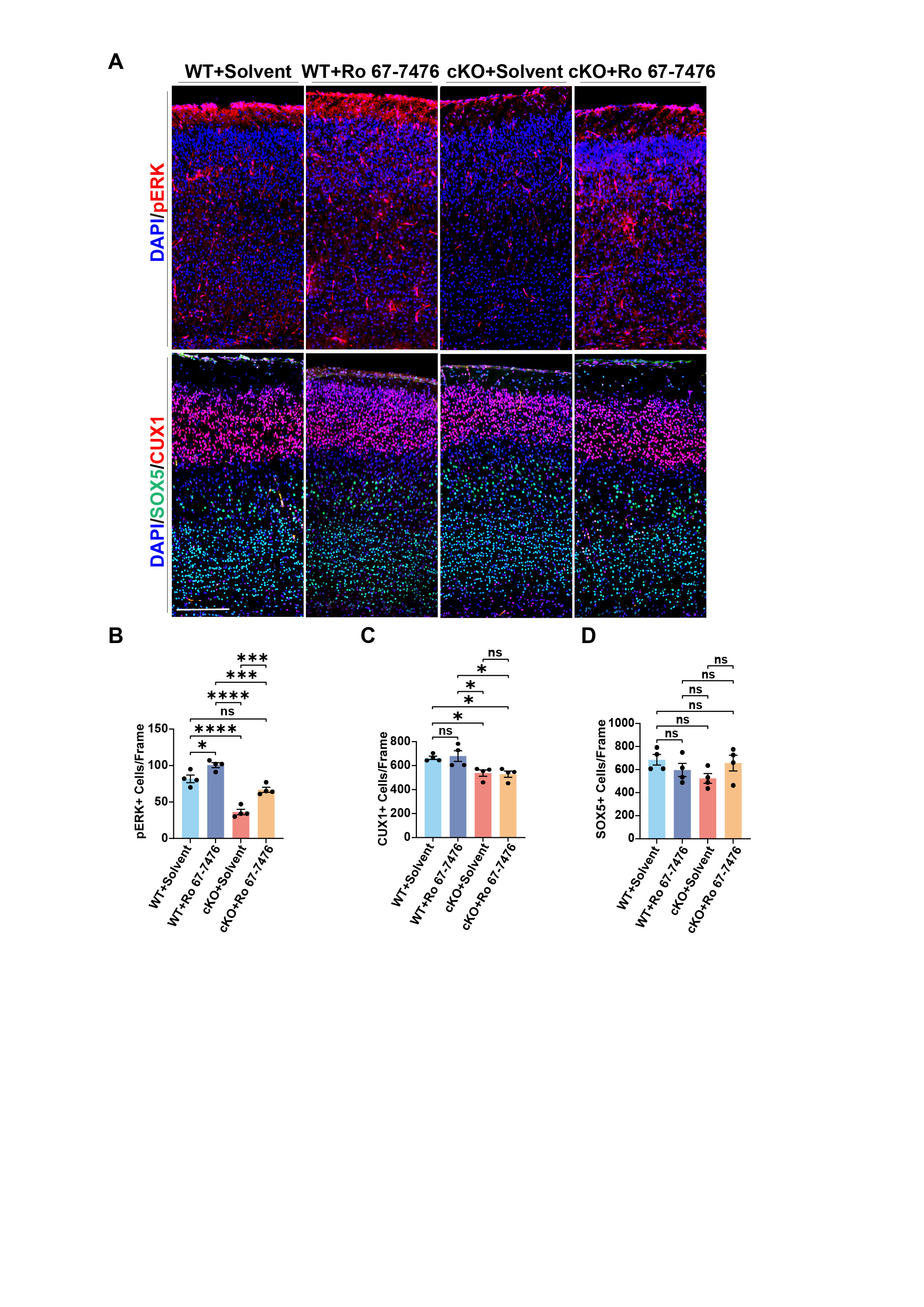


**Figure S11. Effect of pharmacological p-ERK activation on neuronal marker expression in the *Nsd2*-deficient cortex.** (A) Representative immunofluorescence images showing p-ERK and the neuronal markers CUX1 (upper cortical layers) and SOX5 (deep cortical layers) in cortices of vehicle- or Ro 67-7476-treated P3 mice. Scale bar, 200 μm. (B) Quantification of p-ERK^+^ cell density from the experiment in (A). (C, D) Quantification of CUX1^+^ (C) and SOX5^+^ (D) neuronal densities from the experiment in (A).

Data are presented as mean ± SEM; n = 4 biological replicates per group. Statistical significance was determined by one-way ANOVA. *P < 0.05, **P < 0.01, ***P < 0.001, ****P < 0.0001.


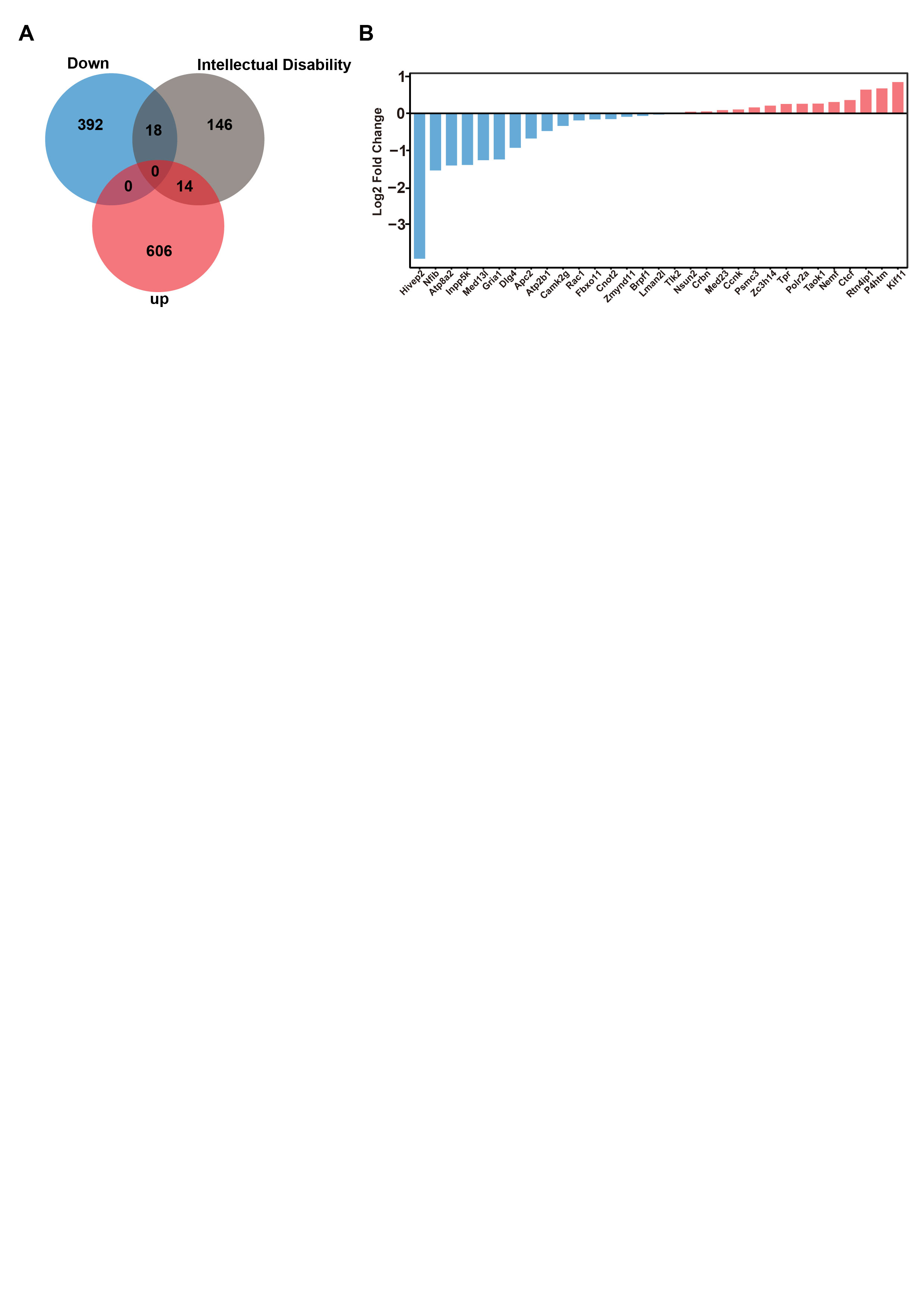


**Figure S12. Transcriptomic analysis reveals an association between *Nsd2* deficiency and intellectual disability.** (A) Venn diagram showing the overlap between differentially expressed genes (DEGs) with human orthologs and intellectual disability–associated genes curated from the Online Mendelian Inheritance in Man (OMIM) database. (B) Histogram depicting the Log2 fold changes of the overlapping DEGs identified in (A).


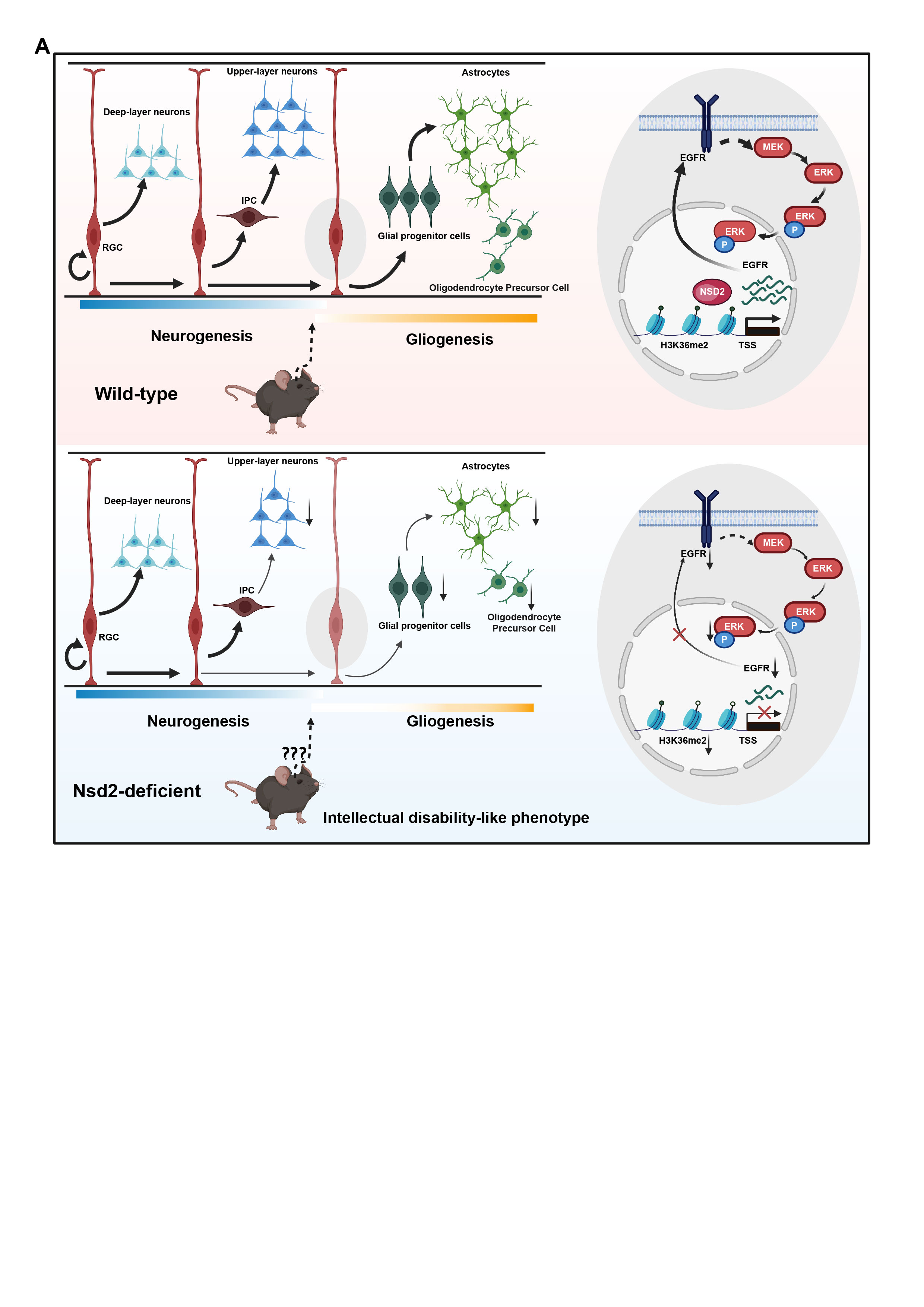


**Figure S13. Schematic diagram of the role of NSD2 in neurogenesis-to-gliogenesis switch.**
